# Oligomer Formation Effects on the Separation of Trivalent Lanthanide Fission Products

**DOI:** 10.1021/acs.inorgchem.4c01272

**Published:** 2024-07-10

**Authors:** Lauren E. Walker, Scott L. Heath, Jun Jiang, Louise S. Natrajan, Francis R. Livens

**Affiliations:** † Department of Chemistry, Faculty of Science and Engineering, The University of Manchester, Manchester M13 9PL, U.K.; ‡ Department of Earth and Environmental Sciences, The University of Manchester, Manchester M13 9PL, U.K.; § AWE, Aldermaston RG7 4PR, U.K.

## Abstract

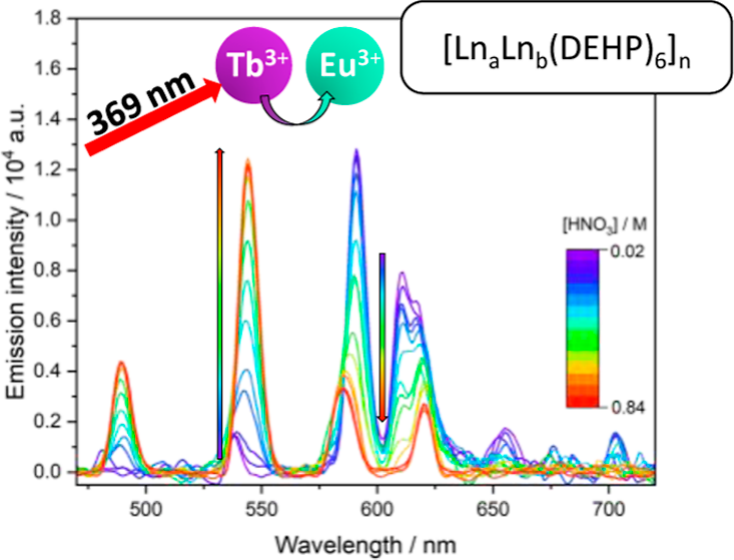

The assessment of trivalent lanthanide yields from the fission of uranium-235 is currently achieved using LN (LaNthanide) resin, di(2-ethylhexyl)orthophosphoric acid immobilized on a solid support. However, coelution of lighter lanthanides into terbium (Tb^3+^) fractions remains a significant problem in recovery of analytically pure fractions. In order to understand how the separation of trivalent lanthanides and yttrium (Ln^3+^) with LN resin proceeds and how to improve it, their speciation with the organic extractant HDEHP must be fully understood under aqueous conditions. A comprehensive luminescence analysis of aqueous solutions of Ln^3+^ in contact with HDEHP, along with infrared spectroscopy, elemental combustion analysis, inductively coupled plasma atomic emission spectroscopy (ICP-AES), and mass spectrometry, was used to indicate that an intermediate species is responsible for the coelution; where similar Ln^3+^ centers (e.g., Eu^3+^ and Tb^3+^) are bridged by the O–P–O moiety of deprotonated HDEHP to form large heteronuclear oligomeric structures with the general formula [Ln_2_(DEHP)_6_]_*n*_. Energy transfer from Tb^3+^ to Eu^3+^ in this structure confirms that lanthanide centers are within 10 Å and was used to propose that the oligomeric [Ln_2_(DEHP)_6_]_*n*_ structure is formed rather than a dimeric Ln_2_(DEHP)_6_ structure. The effect of this speciation on LN resin column elution is investigated using luminescence spectroscopy, confirming that the oligomeric [Ln_2_(DEHP)_6_]_*n*_ species could disrupt regular elution behavior and cause the problematic bleeding of lighter lanthanides (Sm^3+^ and Eu^3+^) into Tb^3+^ fractions. Resin luminescence measurements were used to propose that the bleeding of the organic extractant HDEHP from its solid support causes the formation of the disruptive oligometallic species.

## Abstract 2

The oligomeric structure [Ln_2_(DEHP)_6_]*n* was identified in aqueous systems relevant to separation of trivalent lanthanide fission products using LN resin (HDEHP (di(2-ethylhexyl)orthophosphoric acid) on a polymer support). Luminescence analysis of [Ln_a_Ln_b_(DEHP)_6_]*n* solids and solutions showed a change in the nitric acid concentration at which these oligomeric complexes break up, dependent on the lanthanide present. This altered elution behavior led to the diagnosis that the oligomeric species is responsible for undesired co-elution when using LN resin.

## 1 Introduction

The distribution of fission products is affected by the energy of the neutron causing the fission,^1,2^ and hence analysis of rare earth fission products (trivalent lanthanides and yttrium) is used to support environmental monitoring, nuclear security, radioactive waste treatment, and nuclear safeguards. The main lanthanides of interest are ^141/143/144^Ce, ^147^Nd, ^153^Sm, ^156^Eu, and ^161^Tb.^3^ The activity of Ce isotopes and ^147^Nd can be measured through direct gamma spectroscopy of a sample; however, radiochemical separations are required before gamma spectroscopy of ^153^Sm and ^156^Eu because of their low fission yields (0.158 and 0.015% of atoms/fission, respectively, for ^235^U thermal fission)^2^ coupled with interferences in their spectra.^3^ The quantification of ^161^Tb is particularly problematic because of its low fission yield (8.53 × 10^–5^% of atoms/fission for ^235^U thermal fission), relatively short half-life (6.89 days), and low chemical recovery from the existing radiochemical separations. The gamma spectrum of ^161^Tb is also difficult to resolve because of its low energy gamma emissions and interferences from other nuclides in the detector background.^3^

The almost identical physio-chemical properties of the lanthanides have led to the development of a wide variety of methods for their recovery and separation.^4^ The most popular modern methods, developed in the 1950s, to achieve more efficient separations than those achieved using fractional precipitation and crystallization, make use of solvent extraction and ion exchange technologies.^5,6^ Ion exchange separations are commonly used for high purity analytical separations, and numerous exchange resins and eluents have been employed in the last 50 years for lanthanide intergroup separations.^4,7−9^ The main requirements for lanthanide fission product separations are sufficiently pure lanthanide samples for gamma spectroscopy measurements and rapid separation times before the decay of critically important radionuclides.

Since the 1960s, the most popular method for lanthanide fission product separations has used cation exchange resin with α-HIBA (2-hydroxyisobutyric acid) as an eluent. The main downfall of this technique is poor Tb^3+^ separation yields, resulting in insufficient purification of ^161^Tb for gamma spectroscopy measurements before it has decayed beyond detectable levels.^3^ An improvement to this separation using LN extraction chromatography resin, di(2-ethylhexyl)phosphoric acid (HDEHP) impregnated onto an inert polymer support (40 w/w %), was developed by PNNL (Pacific Northwest National Laboratory) and AWE.^11,12^ Lanthanide sorption and elution has been shown to be dependent on nitric acid concentration; therefore, a gradient was applied to separate neighboring lanthanides with good resolution while keeping the separation time reasonably short (6 h). Results showed an improved separation of lanthanide fractions allowing for ^161^Tb activity to be determined with liquid scintillation counting.^3^

**Figure 1:**
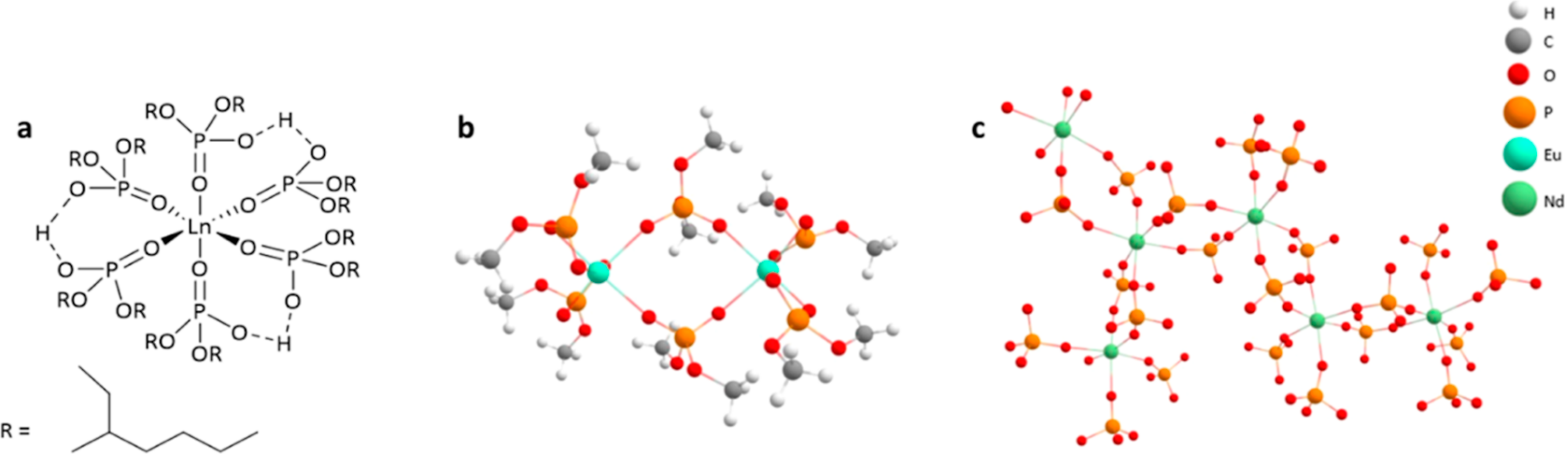
(a) Proposed structure of lanthanide HDEHP complexes on LN resin, three hydrogen-bonded dimers coordinate with the central trivalent lanthanide.^10^ (b) Ln_2_(DEHP)_6_ dimer proposed by Grimes et al.; hydrocarbon chain shortened to methyl groups for clarity.^5^ (c) [Ln_2_(DEHP)_6_]_∞_ polymeric structure proposed by Lumetta et al.^10^ and Gannaz et al.; hydrocarbon chain omitted for clarity.

The final optimized separation utilized two successive LN resin columns, with the second incorporating a modified shortened gradient purely for Tb^3+^ purification. This is a direct result of unexpected bleeding of lighter lanthanides (Eu^3+^ and Sm^3+^) into Tb^3+^ fractions, shown to be dependent on the concentration of Y^3+^ in a sample.^13^ The additional purification step added 3 h to the time-sensitive separation. This coelution effect suggests that the extraction mechanism of LN resin is not as simple as the proposed exchange equilibrium (eq 1),^13^ where a metal center is bound to 3 hydrogen-bonded HDEHP dimers in a pseudo-octahedral manner, depicted in Figure 1a.^10^
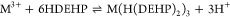
1

The speciation of lanthanides with HDEHP is complex and therefore has been investigated with a variety of techniques, including infrared spectroscopy,^14^ extended X-ray absorption fine structure (EXFAS),^15^ small-angle X-ray scattering (SAXS),^15^ luminescence spectroscopy,^14^ mass spectrometry,^16^ and ^31^P NMR spectroscopy.^16^ The existing literature shows the possibility of differing speciation between HDEHP and the lanthanides from the pseudo-octahedral structure proposed in Figure 1a, where deprotonated ligands (DEHP^–^) bridge Ln^3+^ centers through the O–P–O moiety either in the dimeric form, as shown in Figure 1b (Ln_2_(DEHP)_6_), or in an extended polymeric network, as shown in Figure 1c [(Ln_2_(DEHP)_6_)]_∞_. Grimes et al*.* used time-resolved laser fluorescence spectroscopy (TRLFS) of Eu(III)/HDEHP systems to propose the Ln_2_(DEHP)_6_ species shown in Figure 1b. The absence of the ^5^D_0_ → ^7^F_0_ transition in the emission spectrum supported the existence of a highly symmetrical coordination environment associated with the bridged species at high metal loading ([Ln^3+^]_org_ > 10 mmol L^–1^).^14^ The polymeric bridged structure (Figure 1c) was characterized with single X-ray crystallography of [Nd(DMP)_6_]_∞_, where dimethyl phosphate (DMP) was used as an analogue for HDEHP. The ^4^G_5/2,_^2^G_7/2_ ← ^4^I_9/2_ hypersensitive intra f–f absorption in the diffuse reflectance visible spectrum of [Nd(DMP)_6_]_∞_ crystals and Nd^3+^-saturated HDEHP solutions showed six electronic transitions with a nearly identical splitting pattern consistent with a distorted octahedral structure, indicating very little change in the Nd^3+^ coordination environment with the two different ligands.^10^

EXAFS studies of Ln^3+^ (Ln = Nd, Eu, Yb) complexed with dihexylphosphoric acid (HDHP), used as an analogue for HDEHP, indicated that Ln^3+^ centers have six coordinated O atoms with either three or six distant P atoms. The proposed structures with six distant P atoms were assigned as the O–P–O bridged multimetallic species (Figure 1c), and this was supported by SAXS measurements showing a large polydispersity of the sample.^15^ For Eu^3+^ complexes with three distant P atoms, a structure was proposed, where three DHP^–^ anions coordinate in a monodentate manner to Eu^3+^ in addition to three coordinated water molecules. Hydrogen bonding takes place between the water molecules and two of the uncoordinated oxygen atoms in the DHP^–^ ligand; however, this conclusion disagrees with TRLFS measurements of Eu^3+^/HDEHP systems, which determined there were no coordinating water molecules in the structure.^14^

It is important to note that the majority of current understanding of Ln(III)/HDEHP extractions has developed with respect to understanding the molecular processes involving HDEHP speciation in the TALSPEAK (Trivalent Actinide Lanthanide Separation with Phosphorus-Reagent Extraction from Aqueous Komplexes) process. Under these conditions, the lanthanides are separated by solvent extraction between an organic and aqueous phase with DTPA (diethylenetriaminepentaacetic acid) and lactate buffer at pH 3.6. Therefore, the conditions are not necessarily applicable to those used with an LN resin column. The aqueous conditions under which an LN resin separation is performed (pH lower than 2) will inevitably change the speciation of extracted Ln(III)-HDEHP species. Hence, investigations into the structure of these complexes in an aqueous medium are necessary to fully understand and improve the extraction mechanism using LN resin.

Here, we report evidence for an oligomeric [Ln_2_(DEHP)_6_]_*n*_ species under aqueous conditions using steady state and time-resolved luminescence spectroscopy in combination with ICP-AES (inductively coupled plasma atomic emission spectroscopy), infrared spectroscopy, and mass spectrometry. Using emission spectroscopy, we reveal that mixed metal lanthanide HDEHP species are responsible for coelution that complicates current Ln^3+^ column-based separation technologies. Model and eluent samples show intermetallic energy transfer processes between lanthanide centers (Eu^3+^ and Tb^3+^), implying a heterometallic [Ln_a_Ln_b_(DEHP)_6_]_*n*_ species brings the lanthanides within 10 Å of one another, facilitating intermetallic energy exchange processes. The behavior of these complexes with increasing nitric acid concentration as a model of LN resin column elution establishes that mixed lanthanide species have a large effect on elution behavior; such behaviors have previously remained unconsidered. Together, the data presented herein suggest that leaching of HDEHP from the solid resin support enables the formation of these disruptive species when an Ln^3+^ separation is performed.

## 2 Experimental Section

### 2.1 General Chemicals

All chemicals used were purchased from Merck, Fisher Scientific, or Acros Organics unless otherwise stated. HDEHP was purchased from Alfa Aesar and used without further purification. 50–100 μm LN resin was purchased from TRISKEM International and was used after soaking in 0.1 M HNO_3_ for 24 h.

### 2.2 Lanthanide Stock Solutions

0.005 to 0.31 M lanthanide stock solutions (Eu^3+^, Tb^3+^, Sm^3+^, and Y^3+^) were prepared by dissolving their nitrate salts (Ln(NO_3_)_3_·6H_2_O) in 0.01 M nitric acid. The pH values of the solutions were measured manually using a test cuvette and a Mettler Toledo Seven Compact pH/ION S220 pH probe. Five mM lanthanide stock solutions (Eu^3+^, Tb^3+^, Sm^3+^, and Y^3+^) in deuterated media were prepared by dissolving the nitrate salts in 0.01 M deuterated nitric acid.

### 2.3 Preparation of Ln-HDEHP Solids

An *n*-dodecane solution of HDEHP (2 M, 0.5 mL) was contacted with aqueous Ln(NO_3_)_3_ solution (0.31 M, 1.0 mL) with constant shaking for 2 min. The mixture was centrifuged (7000 rpm) for 10 min, and the aqueous layer removed and discarded. The remaining HDEHP solution was contacted with another 1 mL of Ln(NO_3_)_3_ (0.31 M) and shaken for a further 2 min. The remaining mixture was centrifuged (7000 rpm) for 5 min, and the aqueous phase again was discarded. The solid was suspended in deionized water (0.5 mL) and 2-propanol (0.5 mL) and centrifuged for a further 5 min (7000 rpm). The liquid phase was decanted, and the solid material washed with 5 portions of methanol (25 mL) and dried under reduced pressure (10^–2^ millibar) for 12 h. The elemental composition of the synthesized solids was determined with CHNS analysis using a Flash 2000 elemental analyzer and ICP-OES using a Thermo iCap 6300. Infrared spectroscopy measurements of the solids were recorded on an ALPHA II compact FT-IR spectrometer on a KBr pellet in the range of 4000–500 cm^–1^. Mass spectrometry analysis of lanthanide-HDEHP solids collected from extraction of Ln^3+^ between an aqueous and an organic interface was carried out by the National Mass Spectrometry Centre. Solvent-free MALDI (Matrix Assisted Laser Desorption/Ionization), where a small amount of the solid sample (around 5 mg) is mixed with 10 mg of the DCTB (*trans*-2-[3-(4-*tert*-butylphenyl)-2-methyl-2-propenylidene]malononitrile) matrix, was utilized. The sample mixture was ground in a glass vial using a vortex mixer, with the addition of LiCl, to encourage Li^+^ adduction.

### 2.4 LN resin Loading Experiments

LN resin (0.3 g), preconditioned with 0.1 M HNO_3_ (30 mL), was soaked with Ln(NO_3_)_3_·*n*H_2_O, (*n* = 5,6_,_ 1.12 mM; 9.5 mL) and slowly disturbed on a Stuart Mini See-Saw Rocker (SSM4) for 10 h (25 oscillations/min). The solution was centrifuged (4500 rpm) for 5 min, and the remaining aqueous solution was removed. The soaked resin was spread onto a glass microscope slide and left to dry under atmospheric conditions for 24 h prior to spectroscopic analysis.

### 2.5 Emission Spectroscopy

All room temperature emission data were recorded in quartz cuvettes, 1 cm path length, using an Edinburgh Instruments FLSP920 phosphorimeter equipped with a 450 W steady state xenon lamp, a 5 W microsecond pulsed xenon flash lamp (with single 300 mm focal length excitation and emission monochromators in Czerny Turner configuration), and a red sensitive photomultiplier in Peltier (air cooled) housing (Hamamatsu R928P). All spectra were corrected for the excitation and detector response. Europium(III) luminescence was measured from 560 to 720 nm using 394 nm direct f–f excitation (^5^L_6_ ← ^7^F_0_ transition). Terbium(III) emission was measured from 460 to 640 nm using direct f–f excitation at 369 nm (^5^D_3_ ← ^7^F_6_ transition) and samarium(III) from 520 to 700 nm using 402 nm excitation (^4^G_5/2_ ← ^6^H_3/2_ transition). All measurements were taken using appropriate long-pass filters to minimize scatter and second order effects. Lanthanide-HDEHP solids were measured by placing the sample (∼5 mg) on a glass microscope slide and covering with a quartz coverslip. Spectra used for comparison were recorded using identical settings, and an identical post collection processing was maintained.

Emission spectra were analyzed using OriginProì. Peak positions and heights were measured, and areas were determined through the integration of each peak. Errors associated with the measurements were calculated through the standard deviation of the triplicate repeats.

#### 2.5.1 Ligand Luminescence Titrations

Lanthanide nitrate stock (5 mM; 1.5 mL) was added to 2-propanol (IPA) (1.5 mL). 0.5 mol equiv of HDEHP was added, and the cuvette was shaken for 60 s. The luminescence of the solution was measured without further separation. This was repeated with addition up to 9 mol equiv of HDEHP.

#### 2.5.2 Nitric Acid Luminescence Titrations

Lanthanide nitrate stock (5 mM; 1.5 mL) was added to IPA (1.5 mL) and 6 mol equiv of HDEHP. The solution was shaken for 60 s, and then 70% nitric acid was added in approximately 1 μL aliquots. Luminescence of the solution was measured until no more changes were observed in the emission spectrum of the solution, indicating the complete breakdown of the lanthanide–HDEHP complex.

#### 2.5.3 Lifetime Measurements

Lifetime measurements were recorded using a 5 W xenon microsecond flash lamp. Solution samples were measured at 77 K after flash freezing in NMR tubes in a liquid nitrogen finger dewar. Solid samples were measured at room temperature on a glass microscope slide covered with a quartz coverslip. Europium(III) lifetimes were measured using λ_ex_ = 394 nm at λ_em_ = 590 and 610 nm, and Tb^3+^ lifetimes were measured using λ_ex_ = 369 nm at λ_em_ = 548 nm using multichannel scaling. Lifetimes in deuterated media were measured with lanthanide nitrate stocks made with DNO_3_ and d-IPA.

All lifetime data sets were fitted with exponential decay models using tail fitting starting with the model with fewest terms (monoexponential) and verified by minimization of residuals squared, Chi^2^ and *R*^2^. Instrument response functions (IRFs) were recorded using the procedure outlined by the instrument manufacturer (Edinburgh Instruments), where the excitation and detection wavelengths were matched, and resulting decay was recorded. The contribution of the IRF was removed from the measured lifetimes through the application of fitting parameters only after the instrument response had decayed to background counts.

#### 2.5.4 Stern–Volmer Titrations

Tb(NO_3_)_3_ (20 mM, 0.725 mL) was added to IPA (2 mL) and 3 equiv HDEHP, and the emission of the solution at λ_ex_ = 369 and 394 nm and lifetime of Tb^3+^ (λ_ex_ = 369 nm, λ_em_ = 548 nm) were measured. The procedure was repeated with the addition of Eu(NO_3_)_3_ in 0.1 mol equiv increments up to 1 equiv, the number of moles of Tb(NO_3_)_3_ remained constant, and HDEHP concentration was adjusted to 3 equiv of the overall Ln^3+^ concentration.

Data were analyzed according to the Stern–Volmer model (eq 2), where I is the initial Tb^3+^ emission intensity before the addition of the quencher (Eu^3+^), *I*_0_ is fluorescence intensity in the presence of a quencher; *K*_Sv_ is the Stern–Volmer quenching constant, and [*Q*] is concentration of a quencher.^17^
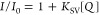
2

## 3 Results and Discussion

### 3.1 Solid Sample Characterization

Trivalent lanthanide speciation with HDEHP was initially investigated through extracting Ln^3+^ (Ln = Sm, Eu, Tb, and Y) into organic solutions of HDEHP and collecting the resulting solid for characterization with IR, mass spectrometry, and elemental analysis.

#### 3.1.1 Infrared Spectroscopy

Lanthanide-HDEHP solids were prepared through centrifuging aqueous lanthanide nitrate salts with an *n*-dodecane HDEHP solution, as described in Section 2.3. Infrared analysis of these solids is shown in Table 1. The characteristic infrared absorption frequencies for organophosphorus acids (RO)_2_P(O)OH were assigned as P=O 1210–1250 cm^–1^, P–O–(H) 1000–1031 cm^–1^, and P–O–(C) 987–1042 or 1014–1060 cm^–1^ by Thomas and Chittenden.^18,19^ On this basis, the following assignments were made for neat HDEHP: P=O, 1223 cm^–1^, P–O–(C), 1010 cm^–1^, and H–O–(P), 1686 cm^–1^. Changes in the infrared stretches are observed upon the complexation of HDEHP by Ln^3+^, indicating a significant change in the (RO_2_)P(O)OH binding moiety. A phosphoryl stretch (P=O) is no longer observed, and the two oxygen atoms that are not bound to alkyl groups become equivalent, resulting in the appearance of an antisymmetric (O–P–O) stretch (ν_a_) observed at 1169–1179 cm^–1^ and symmetric PO_2_ stretch (ν_s_) at 1096–1101 cm^–1^. The P–O–(C) peak is also shifted from 1010 cm^–1^ to around 1030 cm^–1^. The disappearance of the P=O- and H–O–(P)-associated stretches and the appearance of symmetric and antisymmetric PO_2_ stretches indicate deprotonation of the OH group in HDEHP, and hence binding must take place to the lanthanides through the two oxygen atoms unbound to alkyl groups in a bridging manner, as proposed in the structures in Figure 1b,c. The recorded frequencies of the vibrational stretches are in good agreement with those measured by Lumetta et al., who hypothesized the structure depicted in Figure 1c as the coordination between Nd^3+^ and HDEHP.^10^

**Table 1:** IR Assignments

band assignment	HDEHP/cm–^1^	Ln_2_(DEHP)_6_/cm–^1^	[Nd_2_(DEHP)_6_]∞^10^/cm–^1^
P=O	1223		
P–O–(C)	1010	1029–1031	1048/1032/1020
H–O–(P)	1686		
ν_a_ (PO_2_)		1169–1179	1183/1163
ν_s_ (PO_2_)		1096–1101	1095

**Figure 2:**
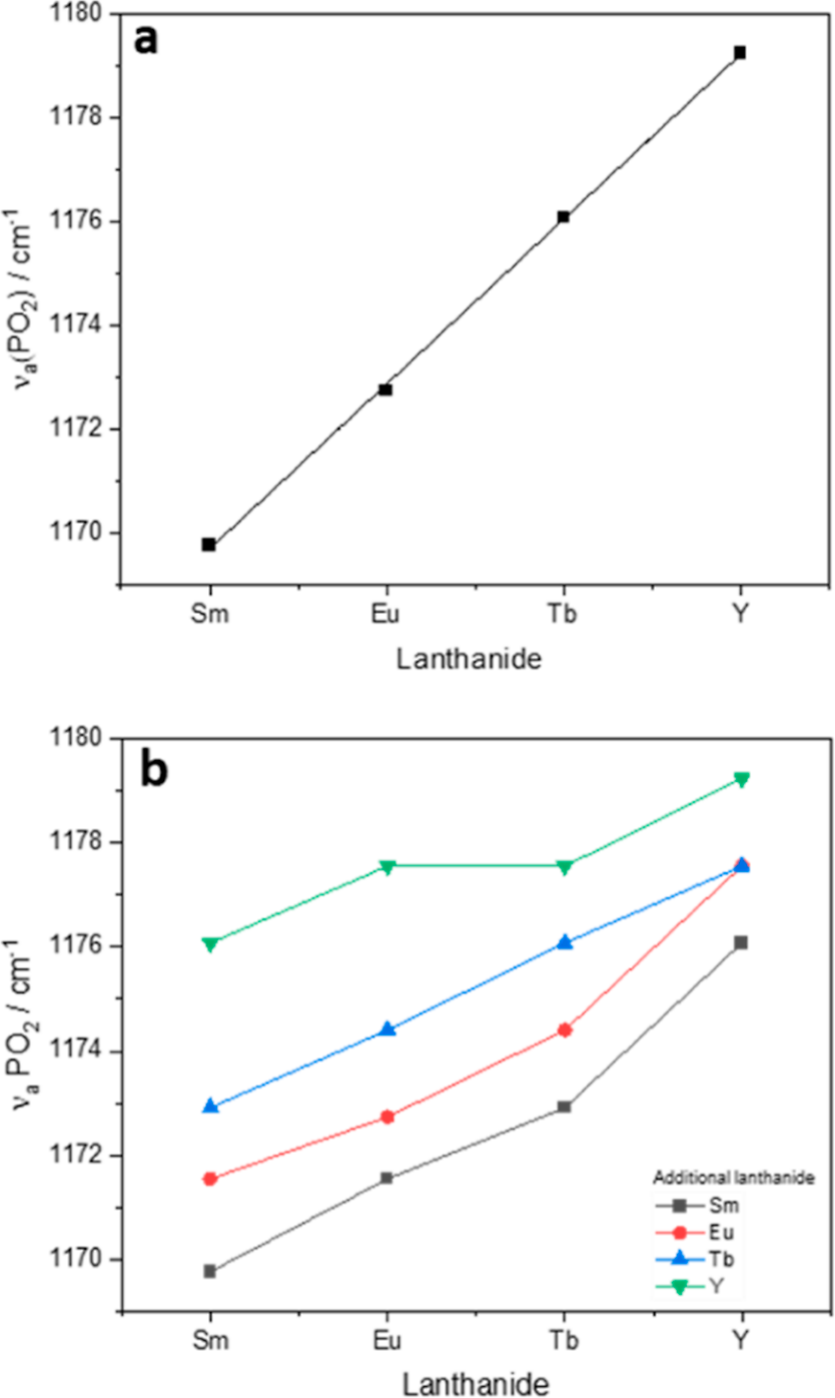
Energy of ν_a_(PO_2_) stretch for (a) Ln(DEHP)_3_ solids collected from the dodecane water interface Ln^3+^ = Sm, Eu, Tb, and Y. (b) Bimetallic lanthanide solids Ln_a_Ln_b_(DEHP)_6_ solids black = Sm^3+^, red = Eu^3+^, blue = Tb^3+^, and green = Y^3+^.

An increase in the energy of the asymmetric PO_2_ stretch across the lanthanide series is observed for the lanthanide solids (Figure 2a). The observed energy increase is in line with the decrease in the 8 and 9 coordinate ionic radius of the trivalent lanthanides across the series, indicating a change in the strength of the PO_2_ bonding environment with the decreasing ionic radius.^20^ The energy of the Nd-HDEHP antisymmetric PO_2_ stretch measured by Lumetta et al. (1163 cm^–1^) is in line with this trend.^10^ Likewise, the same trend is observed for bimetallic solids synthesized with two lanthanides present (Figure 2b). Here, an increase in the frequency of the asymmetric stretch is observed with the addition of a lanthanide with a smaller ionic radius, inferring a change in the strength of the PO_2_ bonding moiety when additional lanthanides are introduced into the structure.

#### 3.1.2 Elemental Analysis

Elemental combustion (CHN) and ICP-AES analysis of Ln-HDEHP solids (see Supporting Information, Table S1) were used to quantify the elemental composition of the solids. The measured Ln^3+^ content of 14% for lanthanide-HDEHP solids supported the conclusion that Ln_2_(DEHP)_6_ speciation results from extraction of Ln^3+^ at an aqueous organic interface; a lower Ln^3+^ content (7%) would be expected for a 1:6 species. Likewise, mixed lanthanide solids contained a total Ln^3+^ content expected from a Ln_a_Ln_b_(DEHP)_6_ species (12–14%), with a slight deviation of the individual lanthanide percentage from values expected of a 1:1 Ln_a_/Ln_b_ sample. Instead, the stronger binding lanthanide (that would elute at a higher concentration) makes up a larger proportion of the solid. As the solids were synthesized with both lanthanides present in excess the Ln^3+^ that has stronger interactions with the HDEHP will preferentially form complexes, hence the deviation from the 1:1 ratio is observed in the samples. This is illustrated by the differing Y^3+^ percentage in mixed samples, where the Y^3+^ content increases with a weaker interacting additional lanthanide in the sample (4.5% for Tb^3+^, 5.2% for Eu^3+^, and 5.7% for Sm^3+^).

#### 3.1.3 Mass Spectrometry Analysis

Analysis of Ln-HDEHP systems using electrospray ionization has previously been utilized for investigations of the structures formed upon portioning with lactate.^16^ MALDI was used for these samples as, unlike ESI, it does not create a large charge distribution allowing for easier analysis of complex mixtures.^21^

**Figure 3:**
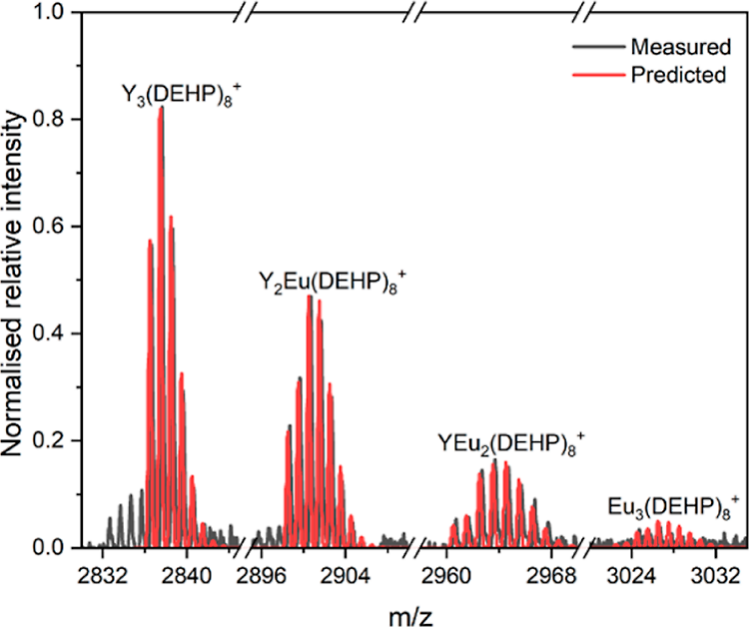
MALDI spectrum for EuY(DEHP)_6_ solid collected from extraction of Ln^3+^ by HDEHP at an aqueous organic interface (black) with predicted molecular ion peaks for Y_3_(DEHP)_8_^+^, Y_2_Eu(DEHP)_8_^+^, (YEu_2_(DEHP)_8_^+^, and Eu_3_(DEHP)_8_^+^ (red).

Analysis of mass spectrometry results suggested that the Ln_2_(DEHP)_6_ samples were complex mixtures, containing fragile metastable species, which decomposed readily as broad, unresolved peaks. Molecular ion peaks were observed for Ln_2_(DEHP)_5_^+^, Ln_2_(DEHP)_6_Li^+^, Ln_3_(DEHP)_8_^+^, Ln_3_(DEHP)_9_Li^+^, and Ln_4_(DEHP)_11_^+^ species for monometallic and heterometallic lanthanide complexes (see Supporting Information Figures S2–S6). In heterometallic samples, molecular ion peaks for different metallic ratios can be observed (1:1, 0:1, 1:0, 2:1 etc.). The peaks corresponding to a species with a higher ratio of stronger binding lanthanide have a larger intensity, indicating that the lanthanides that have the stronger interaction with HDEHP make up more of the sample; this is in line with the ICP-AES analysis of these solids. Figure 3 shows an example of this feature for the Ln_3_(DEHP)_8_^+^ molecular ion peaks. The Y_3_(DEHP)_8_^+^ species has the highest intensity; this declines as Y^3+^ is substituted with Eu^3+^ in the structure. The presence of molecular ion peaks for three and four bridged lanthanide centers indicates that it is likely we are observing fragmentation of an extended polymeric network (Figure 1c). However, the conditions reached during the MALDI experiments may also encourage additional aggregation that would not be found in the solid or solution samples, so we cannot fully conclude whether there is an extended polymeric network present.

Infrared spectroscopy, CHN combustion analysis, and ICP-AES and mass spectrometry analyses indicate that the solid samples from Ln^3+^ extraction with HDEHP at an organic aqueous interface have a 1:3 Ln_2_(HDEHP)_6_ stoichiometry. This analysis alone does not confirm whether a discrete dimer (Figure 1b) or an extended polymeric network (Figure 1c) is the resultant speciation. Further, these solids may not be an appropriate representation of the species that form in the aqueous conditions present on an LN resin column. Luminescence studies were therefore used to confirm whether the Ln^3+^ speciation changes in aqueous conditions and to provide more information on the nature of the DEHP^–^ bridged species.

### 3.2 Luminescence

#### 3.2.1 Emission Investigations of Single Lanthanides with HDEHP

Luminescence of the lanthanides has been used as a probe into their coordination environment as changes in their emission spectra, especially electric dipole induced hypersensitive transitions, can indicate a change in coordination environment.^22^ Luminescence measurements were taken of lanthanide nitrate solutions titrated with HDEHP until no change in luminescence intensity and spectral form was observed; this was compared to luminescence measurements of Ln_2_(DEHP)_6_ solids made by extraction of Ln^3+^ between an aqueous and an organic interface.

**Figure 4:**
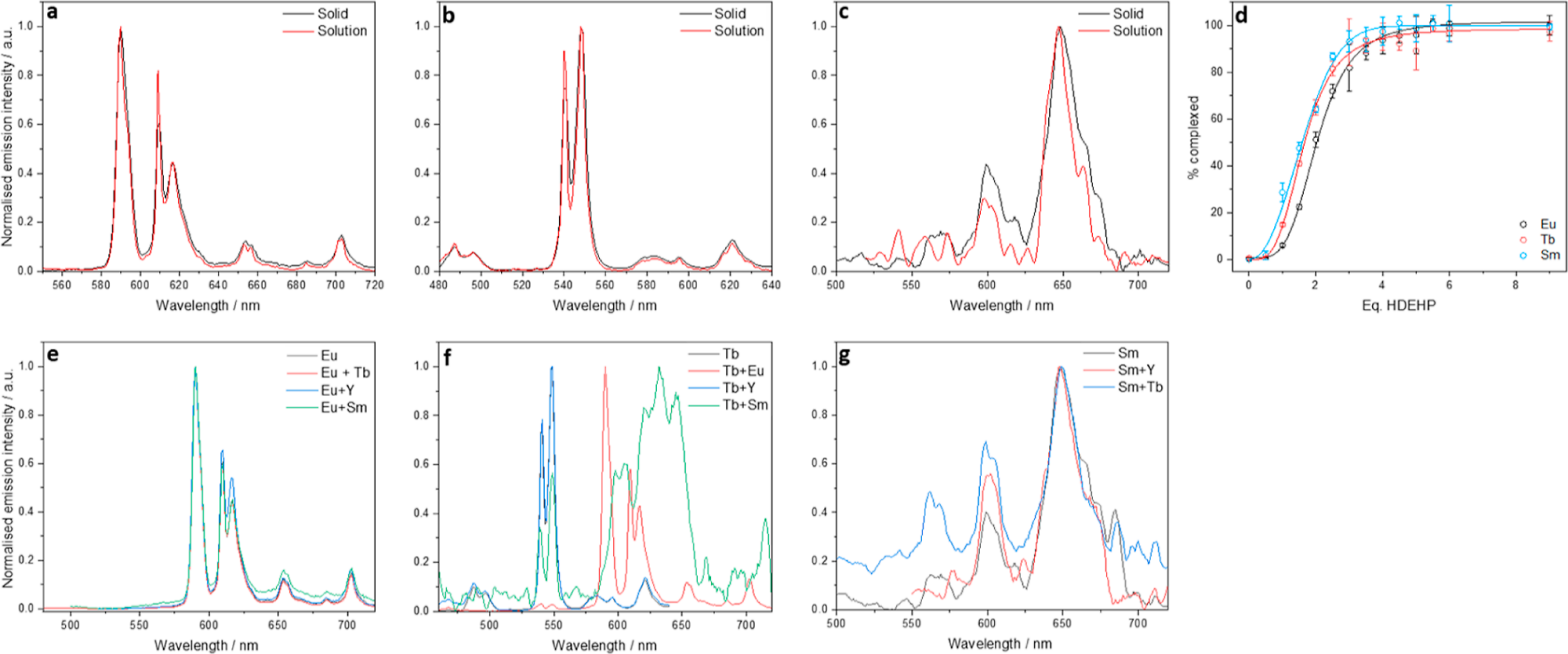
Emission spectrum of (a) Eu(HDEHP) solid vs Eu(HDEHP) solution in H_2_O + IPA, λ_ex_ = 394 nm, (b) Tb(HDEHP) solid vs Tb(HDEHP) in H_2_O + IPA, λ_ex_ = 369 nm, (c) Sm(HDEHP) solid vs Sm(HDEHP) in H_2_O + IPA, λ_ex_ = 402 nm, and (d) percentage of lanthanide complexed at each molar equivalent HDEHP, calculated from relative peak intensities of Ln(NO_3_)_3_ and Ln(HDEHP) species (see Supporting Information, Figure S7). Emission spectrum of (e) EuLn(HDEHP)_6_ bimetallic solids, λ_ex_ = 394 nm Ln = Tb^3+^, Sm^3+^, and Y^3+^, (f) TbLn(HDEHP)_6_ bimetallic solids, λ_ex_ = 369 nm Ln = Eu^3+^, Y^3+^, and Sm^3+^, and (g) SmLn(HDEHP)_6_ bimetallic solids, λ_ex_ = 402 nm Ln = Eu^3+^, Tb^3+^, and Y^3+^.

**Figure 5:**
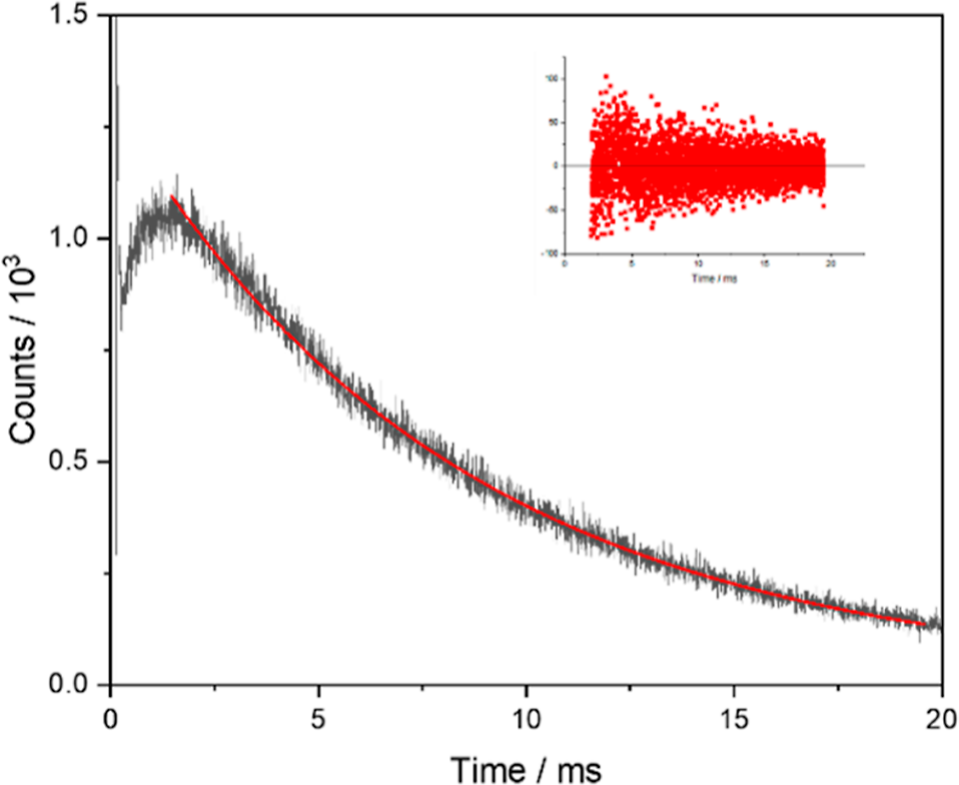
Lifetime decay of EuTb(HDEHP)_6_ solid Eu^3+ 5^D_0_ → ^7^F_1_ emission at 590 nm, with Tb^3+^ excitation via the ^7^F_6_ → ^5^D_3_ transition at 369 nm, in the solid state showing the slow rise in the emission followed by single exponential decay, fitted after subtraction of the IRF. Residual for lifetime fit is shown in the inset, and the rise time fit is given in the Supporting Information (Figure S16).

Emission spectra for single lanthanide-HDEHP solids and solutions are shown in Figure 5a–c. Lanthanides were excited directly into their intra-f-f absorption bands; Eu^3+^ to the ^5^L_6_ state with an excitation wavelength (λ_ex_) of 394 nm;^21^ emission was observed from relaxation into the ^7^F_*J*_ manifold, where *J* = 1, 2, 3, and 4 (Figure 4a). The change in the ratio in intensity of ^7^F_1_/^7^F_2_ peaks from 0.9 for Eu(NO_3_)_3_ to 1.7 for the Eu(HDEHP) species and splitting of the hypersensitive ^7^F_2_ electric dipole transition at 610 nm into two indicates strong perturbation of the Eu^3+^ ion by the ligand field. The Tb^3+^ emission spectra (Figure 4b) were measured following excitation into the ^5^D_3_ excited state with λ_ex_ = 369 nm.^23^ Emission peaks were observed from the ^5^D_4_ → ^7^F_*J*_ manifold where *J* = 6, 5, 4, 3; splitting of the hypersensitive ^7^F_5_ transition at 550 nm was observed. Emission of Sm^3+^ was observed after excitation to the ^4^G_5/2_ state with λ_ex_ = 402 nm (Figure 4c).^23^ The lower intrinsic quantum yield for Sm^3+^ luminescence resulted in difficulties resolving the emission peaks from the baseline, yet the ^4^G_5/2_ → ^6^H_*J*_*J* = 5/2, 7/2, 9/2 transitions could be observed upon subtraction of the baseline.

The emission spectra of the extracted solids and aqueous solutions of Ln-HDEHP were indistinguishable, suggesting that the Ln_2_(DEHP)_6_ speciation observed in Ln-HDEHP solids is the prevailing species in aqueous solutions. Titrations of aqueous lanthanide salts with HDEHP (see Supporting Information Figure S7) further predicted an empirical formula of Ln(DEHP)_3_ as the changes in intensity of the Eu^3+7^F_2_, Tb^3+7^F_5_, and Sm^3+6^H_9/2_ peaks, displayed in Figure 4d, plateau around the addition of 3–4 mol equiv HDEHP, with clear isosbestic points showing only the presence of the Ln(DEHP)_3_ and Ln(NO_3_)_3_ species in the titration (see Supporting Information, Figure S7).

#### 3.2.2 Emission Investigations of Mixed Lanthanides with HDEHP

Lanthanide emission spectra were recorded for solid samples prepared with two Ln^3+^ ions (Ln = Sm, Eu, Tb and Y) in a 1:1 ratio as detailed in Section 2.3. The Eu^3+^ emission (λ_ex_ = 394 nm) spectrum, displayed in Figure 4e, is unchanged in each respective mixed lanthanide solid, displaying identical peak centers, relative intensities and ^7^F_1_/^7^F_2_ ratios of 1.7, suggesting the same local Eu^3+^ coordination environment remains throughout the mixed lanthanide samples. Contrastingly, differences are observed in the emission spectra of Tb^3+^ in Figure 4f (λ_ex_ = 369 nm). There is no change in the Tb^3+^ emission in the mixed Tb^3+^ and Y^3+^ solid. However, in samples containing Eu^3+^ or Sm^3+^, quenching of the ^7^F_5_ transition at 545 nm is observed along with new emission peaks, a broad peak around 600–650 nm for Sm^3+^ samples and a number of sharp peaks from 590 to 720 nm for Eu^3+^ samples. These new emission peaks can be attributed to weak Sm^3+^ emission and the ^7^F_*J*_ (*J* = 1, 2, 3, and 4) Eu^3+^ emission peaks, respectively. The observation of Eu^3+^ and Sm^3+^ emission when exciting Tb^3+^ indicates a route exists in the mixed lanthanide samples in which energy transfer can take place between trivalent lanthanides, where the excited state donor (here, Tb^3+^) and lower energy acceptor emissive levels (Sm^3+^ and Eu^3+^) are energetically matched facilitating intermetallic energy transfer processes.^24^ This is supported by the spectroscopic investigations of the Sm^3+^ samples (λ_ex_ = 402 nm), where the emission remains unchanged with Y^3+^, but is enhanced in intensity in the presence of Tb^3+^. Titrations of 1:1 mixed lanthanide nitrate solutions with HDEHP displayed a plateau around 3–4 mol equiv HDEHP (see Supporting Information, Figure S8), and all emission spectral results of mixed lanthanide solids were reproducible in solution, indicating that the Ln_a_Ln_b_(DEHP_6_) speciation persists in mixed lanthanide solution samples as a discrete molecular heterometallic complex.

#### 3.2.3 Kinetic Decay Profiles

Quenching of lanthanide excited states typically takes place through the harmonics of closely diffusing O–H oscillators; this quenching is greatly reduced in deuterated solvents, which have a negligible Franck–Condon overlap between the Ln^3+^ excited state and harmonics of the O–D oscillations. This effect is exploited to quantify *q*, the number of inner sphere solvent molecules that can be associated with the lanthanide ion. For complexes with fewer than three coordinated solvent molecules, the value of *q* is best obtained from eq 3.^25^
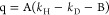
3where *k*_H_ and *k*_D_ are the measured rate constants for the luminescence decay in regular and deuterated solvents, respectively, and *A* and *B* are experimentally determined constants for a particular lanthanide. The solvent mixture in this study deviates from that used by Beeby et al. in the calculations of the constants *A* and *B*, introducing additional uncertainty in the calculation of *q*.^25^ The measured lifetimes and corresponding number of inner sphere water molecules (Table 2) suggest an inner sphere hydration number of 1 which is higher than the value of 0 reported by Grimes et al.^13^ This is likely due to the fact the sample was measured in water/isopropyl alcohol mixtures, whereas other similar studies have been conducted in organic solvents. The suggested coordination number of 7 is low for lanthanide complexes, which are typically 8 or 9 coordinate,^23,26^ but the considerable steric bulk provided by the six branched hydrocarbon chains should sufficiently stabilize the Ln^3+^ center.

**Table 2:** Mixed Lanthanide Lifetimes in a 1:1 v/v Ratio Mixture of H_2_O/Isopropyl Alcohol and D_2_O/*d*-Isopropyl Alcohol^a^

lanthanide	τ H_2_O/ms	τ D_2_O/ms	*q*
Eu	0.6	1.5	0.8
Eu (+Tb)	0.5	1.4	1.4
Eu (+Sm)	0.5	1.4	1.3
Eu (+Y)	0.6	1.6	1.0
Tb	0.8	1.2	1.4
Tb (+Eu)	0.3	0.3	2.8
Tb (+Y)	0.8	1.1	1.3

a The number of inner sphere water molecules (*q*) was calculated using eq 3. *q* calculated for Tb (+Eu) will be artificially high due to intermetallic energy transfer to Eu^3+^. All original traces and resulting fits can be found in Supporting Information, Figures S9–S15.

The shortening of the Tb^3+^ lifetime from 0.8 to 0.3 ms for TbEu(DEHP)_6_ complexes indicates that in a Tb^3+^/Eu^3+^ mixed sample, a route exists in which the Tb^3+^ excited state can be rapidly depopulated with energy transfer into the Eu^3+^ exited state. Energy transfer between metals, often bound in a heterometallic complex, is correlated with changes in luminescence lifetime of the excited state when an energetic pathway from the donor excited state to the acceptor results in a reduction in lifetime of the donor.^24^ The time scale of this energy transfer can be observed through the lifetime of the 590 nm ^5^D_0_ → ^7^F_1_ Eu^3+^ emission when indirectly excited through the Tb^3+5^D_3_ excited state (λ_ex_ = 369 nm). This kinetic profile (displayed in Figure 5) shows a rise corresponding to the population of the Eu^3+5^D_0_ excited state (≈1 ms) before decaying exponentially. The lifetime of Tb^3+^ emission in this system without Eu^3+^ matches the rise time of the Eu^3+^ lifetime (≈1 ms), suggesting that there is a direct energy transfer from the Tb^3+5^D_4_ excited state into the Eu^3+5^D_0_ excited state.^27^

#### 3.2.4 Stern–Volmer Titrations

**Figure 6:**
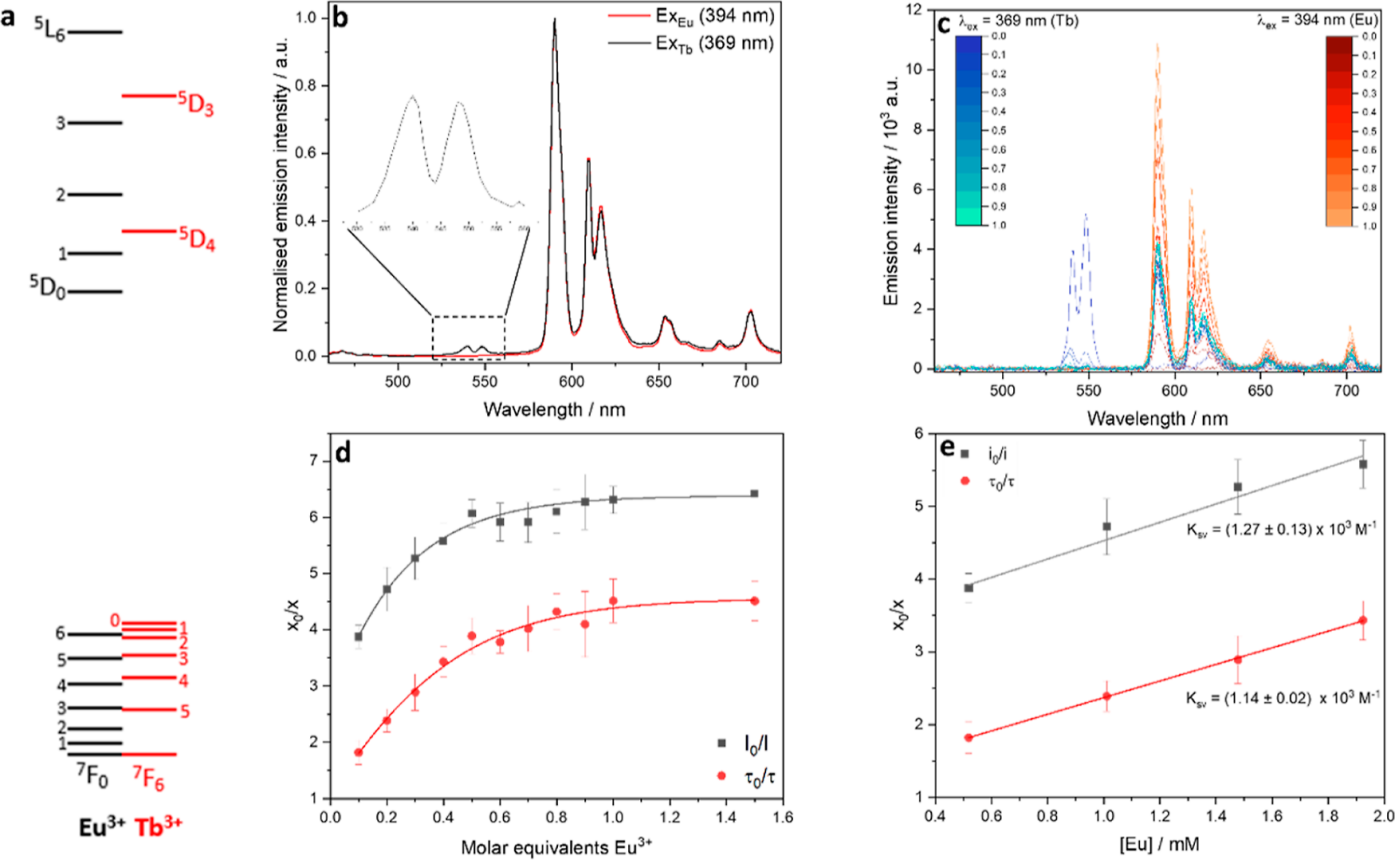
(a) Electronic energy levels for Eu^3+^ and Tb^3+^; the excited states are close in energy, allowing for transfer from the Tb^3+5^D_3,4_ excited states into the Eu^3+5^D_*J*_*J* = 0,1,2,3 excited states. (b) Emission spectrum of TbEu(HDEHP)_6_ solid, measured at λ_ex_ = 369 nm (black) and λ_ex_ = 394 nm (red), normalized. (c) Emission spectra recorded for SV quenching experiments, λ_ex=_ 369 nm (blue) and λ_ex_ = 394 nm (red), increasing molar equivalents of Eu^3+^ in the sample up to 1:1 ratio of Ln metals. (d) Stern–Volmer plots of *I*_0_/*I* (gray squares), where *I*_0_ is initial intensity of the Tb^3+7^F_5_ emission at 545 nm, λ_ex_ = 369 nm, and I is the intensity upon addition of 0.1–1.5 mol equiv of Eu^3+^ and τ_0_/τ (red circles), where τ_0_ is the initial lifetime of the Tb^3+5^D_4_ → ^7^F_5_ emission peak and τ is the lifetime upon addition of 0.1–1.5 mol equiv of Eu^3+^. (e) Linear region of Stern–Volmer titrations, upon addition of 0.1–0.4 mol equiv of Eu^3+^. Quenching efficiency, *K*_SV,_ calculated from gradient of the linear fit.

Energy transfer from Tb^3+^ to Eu^3+^ is well documented with examples in glasses,^28,29^ inorganic compounds,^30^ and in solution;^31^ energy is transferred from the ^5^D_4_ excited state into the ^5^D_0_ Eu^3+^ excited state (Figure 6a). In many reported cases of Tb^3+^ to Eu^3+^ energy transfer, a detailed mechanism through which this takes place is not clear; different studies have attributed the interaction from Tb^3+^ to Eu^3+^ as electric dipole (ED–ED),^32,33^ electric dipole-electric quadrupole (ED-EQ),^34^ or exchange interactions.^35^ It is likely that the energy transfer mechanism will depend on the coordination environment of each sample. Luo et al. determined that the ED–ED interaction is the energy transfer mechanism in an orthophosphate system, as the Tb^3+ 5^D_4_ → ^7^F_*J*_ (*J* = 6–4) and Eu^3+ 5^D_0_ ← ^7^F_1_ transitions are induced electric dipole allowed.^27^ Solarz reported a limiting distance for this ED–ED quenching to be 9.96 Å.^36^ For energy transfer to be feasible in the systems under study here and the cause of quenching of Tb^3+^ emission as in Figure 6b, the two Ln^3+^ centers must be located within this distance (9.96 Å). A single crystal X-ray diffraction study of the related complex [Nd_2_(DMP)_6_], containing the same O–P–O bridged moiety proposed for lanthanide-HDEHP complexes, contained Nd–Nd distances of 5.90 Å.^14^ An equivalent O–P–O bridge in the mixed Ln-HDEHP systems would feasibly bridge the two Ln^3+^ centers bringing them close enough to allow for this observed energy transfer.

**Figure 7:**
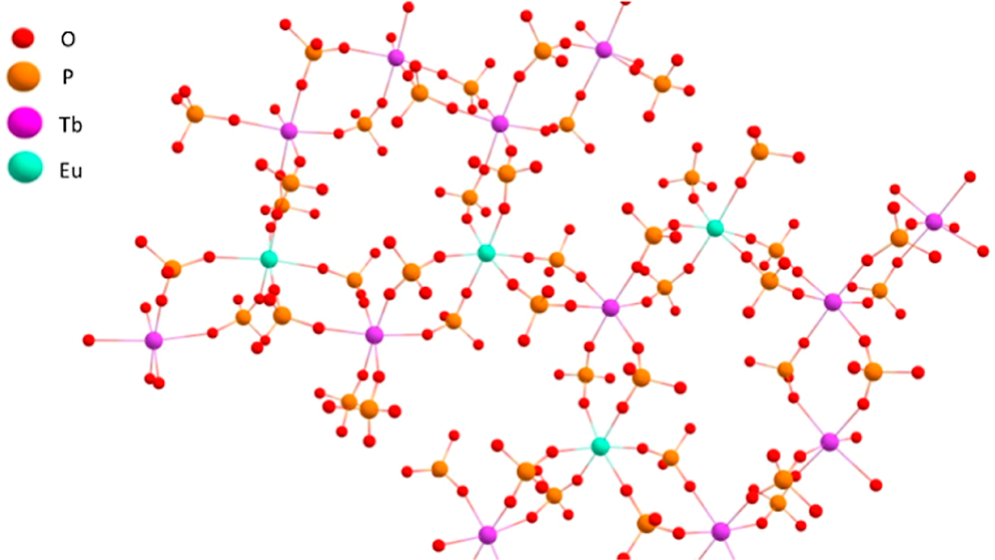
Illustration of a possible chemical structure for [EuTb(DEHP)_6_]_*n*_ formed in the reaction of a 1:1 mixture of Eu(NO_3_)_3_ and Tb(NO_3_)_3_ with 3 equiv HDEHP in a 1:1 v/v mixture of IPA and H_2_O. The illustration was generated using the single crystal X-ray data from ref (14) and modified in ChemCraft to change the lanthanide ions to Eu^3+^ and Tb^3+^ (from Nd^3+^) according to the ratios measured using ICP-OES.

Stern–Volmer titrations were used to discern whether the energy transfer is the result of bridged discrete dimers (Figure 1b) or an extended polymeric network (Figure 1c), as each structure should display characteristic quenching behavior that is stoichiometry-dependent. The emission spectra recorded for the luminophore donor (Tb^3+^, λ_ex_ = 369 nm) and quencher (Eu^3+^, λ_ex_ = 394 nm) in titrations with increasing molar equivalents of quencher are plotted in Figure 6c. The Eu^3+^ emission increases linearly with increasing Eu^3+^ concentration when excited into the ^5^L_6_ excited state with λ_ex_ = 394 nm, whereas excitation via Tb^3+^ into the Tb^3+5^D_3_ excited state at λ_ex_ = 369 nm results in a plateau in intensity change after the addition of 0.4–0.5 equiv Eu^3+^. This plateau demonstrates that Eu^3+^ emission observed at λ_ex_ = 369 nm is limited by the concentration of Tb^3+^ in the sample. Figure 6d shows Stern–Volmer plots of *I*_0_/*I* and τ_0_/τ, where *I*_0_ is the intensity of the Tb^3+7^F_5_ emission at 548 nm, τ_o_ is Tb^3+^ lifetime before the addition of quencher, and *I* and τ are the intensity and lifetime with each subsequent Eu^3+^ addition. The gradient of the linear region of these plots (Figure 6e) was used to calculate the quenching efficiency of Tb^3+^ by Eu^3+^, *K*_SV_ = (1.27 ± 0.13) × 10^3^ M^–1^, and *K*_SV_ = (1.14 ± 0.02) × 10^3^ M^–1^. The complete quenching of Tb^3+^ emission after the addition of 0.4–0.5 equiv Eu^3+^ suggests this is the molar ratio at which all Tb^3+^ ions are within 9.96 Å of an Eu^3+^ ion.^36^ This would not be the expected quenching behavior if dimeric Ln_2_(DEHP)_6_ complexes were forming; instead a linear increase in quenching with increasing Eu^3+^ concentration would be observed, as the probability of forming a TbEu(DEHP)_6_ bimetallic species would increase with Eu^3+^ concentration until Eu^3+^ was in large excess. The plateau at 0.5 equiv also does not match the quenching expected from a fully polymeric network [EuTb(DHEP)_6_]_∞_; in this structure, it would be possible for three Eu^3+^ ions to be within 9.96 Å of a single Tb^3+^, so a plateau in quenching around 0.33 mol equiv would be expected. It was determined that the quenching behavior instead matches that which would be expected of an oligomeric bridged network [TbEu(DEHP)_6_]_*n*_ (Figure 7). In this structure, three Tb^3+^ atoms could still be quenched by a single Eu^3+^ in the center of the structure; however, Tb^3+^ ions at the edge of the structure can only be quenched by one or two Eu^3+^ ions; hence, the plateau at the slightly higher 0.5 mol equiv Eu^3+^ is observed.

Quenching behavior was comparable between solid and solution samples, with a strong quenching of Tb^3+^ emission observed with the addition of 0.1 equiv Eu^3+^ and a plateau in quenching after the addition of 0.4–0.5 equiv Eu^3+^, further indicating that speciation is maintained between solid and solution samples. Taking the quenching behavior into account along with all luminescence, IR, CHN, and ICP-AES data, it is likely that the speciation between HDEHP and the lanthanides in aqueous solution is an oligomeric structure [(Ln_2_(DEHP)_6_]_*n*_ similar to that illustrated in Figure 7. The effect of this structure on elution from an LN resin column was investigated to discern whether this can cause the coelution of Eu^3+^ and Sm^3+^ into Tb^3+^ fractions.

#### 3.2.5 Nitric Acid Titrations

**Figure 8:**
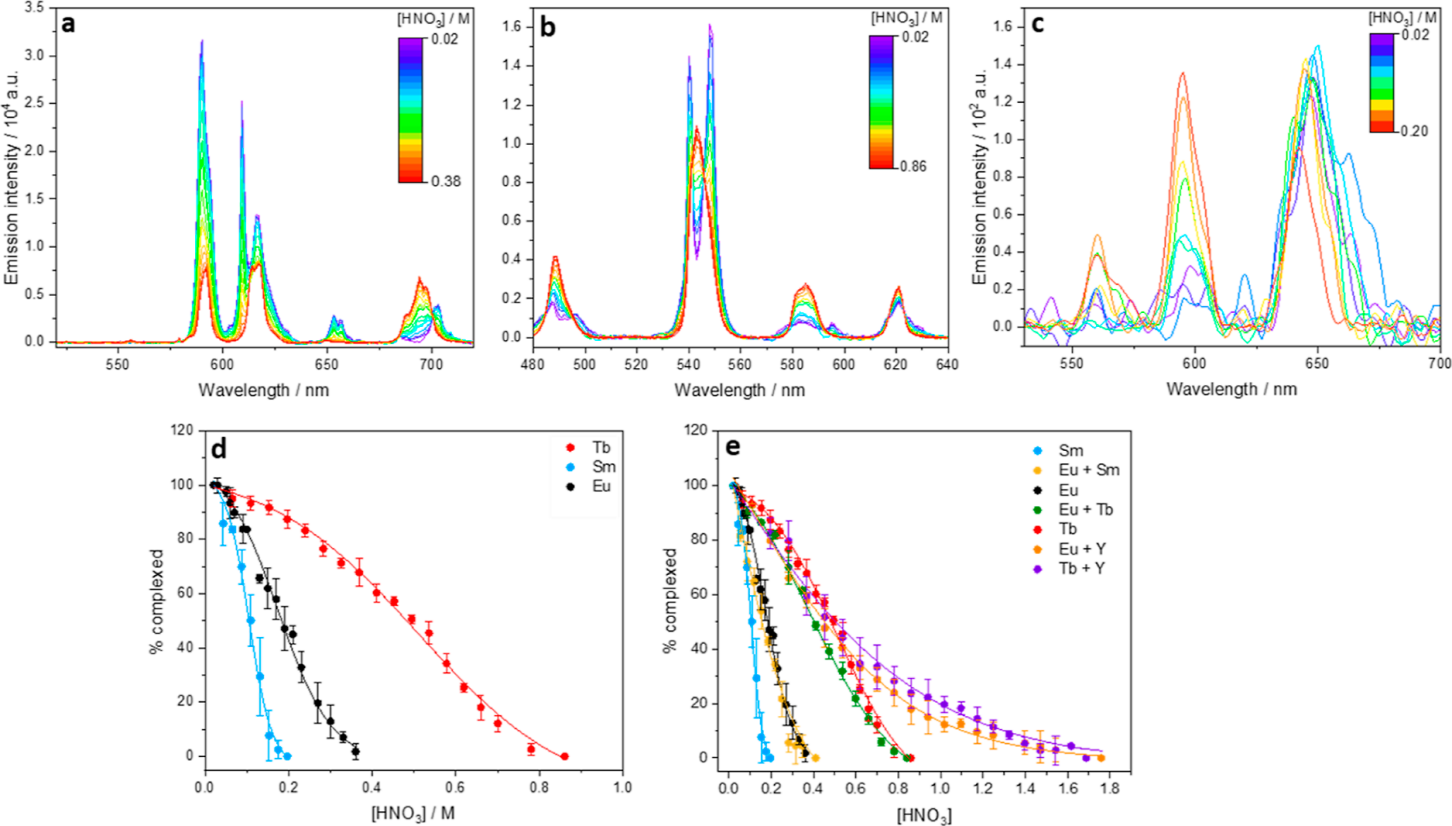
Luminescence emission spectrum of 5 mM: (a) Eu_2_(DEHP)_6_ λ_ex_ = 394 nm initial [HNO_3_] = 0.02 M, final [HNO_3_] = 0.38 M increasing in 0.02 M increments. (b) Tb_2_(DEHP)_6_ λ_ex_ = 369 nm initial [HNO_3_] = 0.02 M finial [HNO_3_] = 0.86 M increasing in 0.04 M increments. (c) Sm_2_(DEHP)_6_ λ_ex_ = 402 nm initial [HNO_3_] = 0.02 M final [HNO_3_] = 0.20 increasing in 0.02 M increments. All measured in a 1:1 v/v H2O/IPA solvent. (d) Percentage complexed of each lanthanide at a nitric acid concentration. (e) Percentage complexed of each lanthanide in mixed lanthanide HDEHP solutions with the increasing nitric acid concentration. It is calculated through the change in the peak area and intensities of Eu^3+5^D_0_ → ^7^F_2_ emission peak, Tb^3+5^D_4_ → ^7^F_5_ emission peak, and Sm^3+4^G_5/2_ → ^6^H_9/2_ emission peak with titrations of 1:1 Ln_a_Ln_b_(DEHP)_6_ in 1:1 v/v IPA + H_2_O with nitric acid (see Supporting Information, Figure S17). The spectra have been rescaled (using the tool in OriginPro) to allow for easy visual comparison.

The elution of HDEHP species from an LN resin column was modeled through titrations of the proposed [Ln_2_(DEHP)_6_]_*n*_ solutions with concentrated nitric acid. The changes in emission spectra with increasing nitric acid concentrations show the change of Ln^3+^ speciation from [Ln_2_(DEHP)_6_]_*n*_ complexes to aqueous nitrate (Figure 8). The isosbestic points, observed in the Eu^3+^ and Tb^3+^ emission spectra, indicate only the [Ln_2_(DEHP)_6_]_*n*_ and Ln(NO_3_)_3_ species are present in solution with no observable intermediates in the dissociation of the complex on the time scale of the luminescence experiment (ms). The intensity of the emission from each species was calculated through peak deconvolution, allowing the relative concentration of each lanthanide species to be modeled at each nitric acid concentration (Figure 8d). The relative behavior of each lanthanide matched the observed behavior on an LN resin column; Sm^3+^ elutes first with complete breakup of the HDEHP complex at 0.2 M, Eu^3+^ at 0.4 M, and Tb^3+^ at 0.9 M. Y^3+^ elution could not be modeled in this way as it is optically silent. Having confirmed that the above conditions were a suitable model for a LN resin column, mixed lanthanide solutions were then titrated with nitric acid and modeled in the same manner.

The percentage of lanthanide complexed at each nitric concentration is plotted in Figure 8e. Here, we observed a large change in the elution behavior of the Ln^3+^ dependent on the additional Ln^3+^ in solution. For example, observing the elution of Eu^3+^ in different mixed samples; with Sm^3+^, which elutes at lower nitric acid concentrations, the elution profile matches that of Eu^3+^ alone (0.4 M). With Tb^3+^, the Eu^3+^ ion elutes at a nitric acid concentration where Tb^3+^ would elute alone (0.85 M), and with Y^3+^, this concentration is raised even further (1.8 M). The differences in the elution behavior for the mixed lanthanide samples were attributed to the bridging O–P–O moiety, linking the different lanthanides in the structure. Infrared spectroscopic data of the mixed metal solids showed a change in the Ln–O–P bond strength when different lanthanides were introduced into the complex structure; this will likely change the nitric acid concentration at which the O–P–O moiety is protonated, and the lanthanide is released into solution from the oligomeric HDEHP complex. There is a discrepancy between the amount of coelution observed in these aqueous measurements and the real coelution observed on an LN resin column, where only a small percentage of Eu^3+^ and Sm^3+^ are present in the Tb^3+^ fraction (with the amount of Eu^3+^ and Sm^3+^ in the Tb^3+^ fraction dependent on the concentration of Y^3+^ in the separation).^3,10^ The polymeric support of LN resin will change the way that HDEHP can bind to the lanthanides, so luminescence measurements were taken of lanthanide soaked LN resin to compare speciation on the resin and in aqueous solution.

#### 3.2.6 LN Resin Speciation

To investigate the possibility of bridged lanthanide structures forming on the LN resin, luminescence measurements were taken of samples of 0.3 g of LN resin (50–100 μm) soaked with 9.5 mL of 1:1 solution of 1.12 mM Eu(NO_3_)_3_ and Tb(NO_3_)_3_. The soaked resin was centrifuged, and the remaining lanthanide solution removed, whereupon the resin was spread onto a microscope slide and dried under atmospheric conditions. When exciting into the Eu^3+5^L_6_ excited state, λ_ex_ = 394 nm, the Eu^3+^ emission ^7^F_1_/^7^F_2_ ratio (0.23) was significantly different to the ratio for the [Eu_2_(DEHP)_6_]_*n*_ complex (1.70), which indicates a change in symmetry around the Eu^3+^ ion to a highly asymmetric environment when bound to the resin.^23^

**Figure 9:**
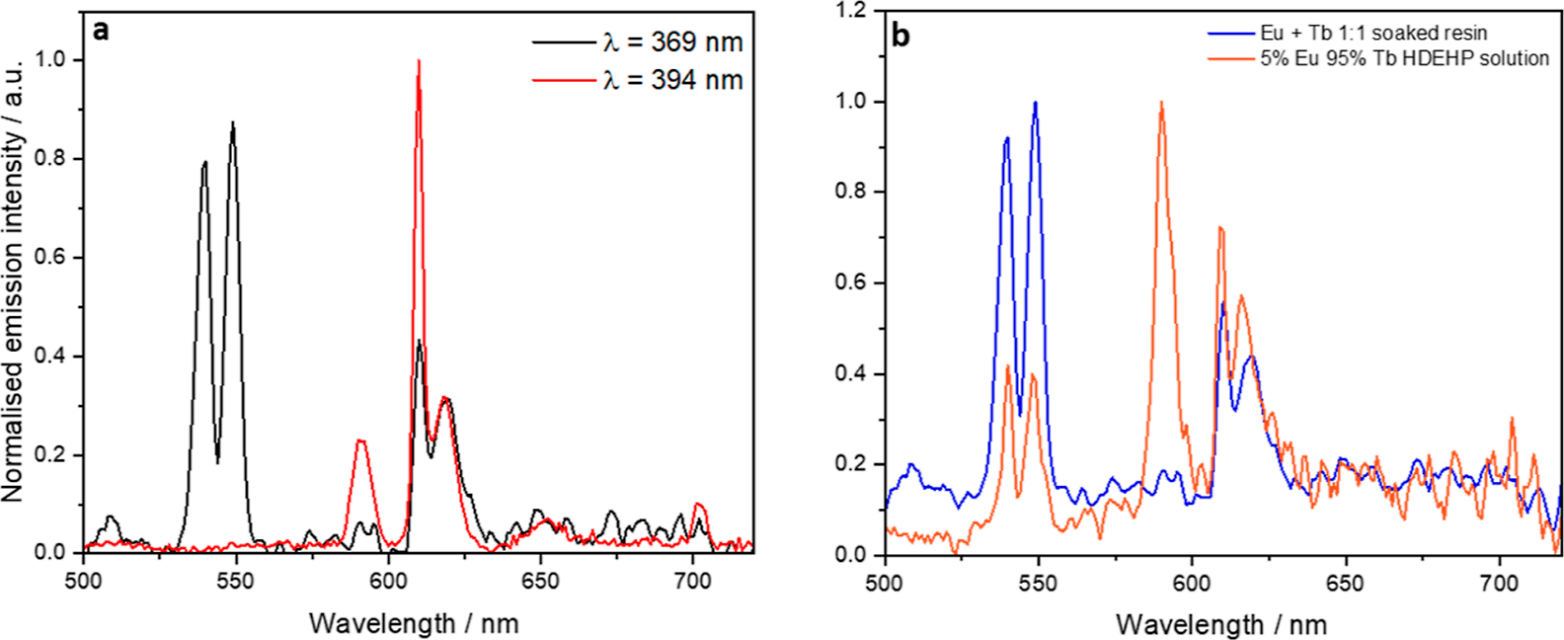
(a) Emission spectrum of LN resin soaked in 9.95 mL 1:1 Tb(NO_3_)_3_ + Eu(NO_3_)_3_ (1.12 mM) with Tb^3+^ excitation at 369 nm (black) and Eu^3+^ excitation at 394 nm (red). (b) Emission spectrum of LN resin soaked with 1:1 Tb(NO_3_)_3_ + Eu(NO_3_)_3_ excited at 369 nm (blue) compared to a 5% Eu^3+^ 95% Tb^3+^ nitrate solution saturated with HDEHP excited at 369 nm (orange). The spectra have been rescaled (using the tool in OriginPro) to allow for easy visual comparison.

It is likely that the HDEHP on the resin cannot occupy all the binding sites of Ln^3+^ sorbed to the resin, and instead only one deprotonated HDEHP molecule occupies two binding sites of the lanthanides and the remaining coordination sphere is likely inner sphere water molecules. Excitation into the ^5^D_3_ Tb^3+^ excited state (λ_ex_ = 369 nm) resulted in the observation of Tb^3+^ emission peaks for the ^5^D_4_ → ^7^F_*J*_ (*J* = 6, 5) transitions and Eu^3+^ emission peaks for the ^5^D_0_ → ^7^F_*J*_ (*J* = 1, 2) transitions (Figure 9a). The appearance of Eu^3+^ emission at this excitation wavelength indicates that a route still exists in the resin samples for energy transfer from the Tb^3+5^D_4_ excited state to the Eu^3+5^D_0_ excited state.

Two notable differences can be seen in the emission resulting from energy transfer on the resin versus in the aqueous HDEHP solutions. The splitting of the hypersensitive ^7^F_2_ Eu^3+^ emission peak at 610 nm is different if Eu^3+^ is excited via the ^5^L_6_ Eu^3+^ excited state (λ_ex_ = 394 nm) compared to excitation via the ^5^D_3_ Tb^3+^ excited state (λ_ex_ = 369 nm). Direct Eu^3+^ excitation results in a splitting ratio (*I*_610_/*I*_618_) of 3.2 whereas excitation through energy transfer from Tb^3+^ yields a splitting ratio of 1.4, which matches the splitting of the ^7^F_2_ peak previously measured in the emission spectra of [Eu_2_(DEHP)_6_]_*n*_ and [EuTb(DEHP)_6_]_*n*_ species. The intensity of Tb^3+^ emission is larger than the intensity of Eu^3+^ emission with direct Tb^3+^ excitation (λ_ex_ = 369 nm) for emission measurements of the resin. This is a large change from the almost complete quenching of Tb^3+^ emission with direct Tb^3+^ excitation (λ_ex_ = 369 nm) for a 1:1 EuTb(DEHP)_6_ complex measured for the proposed bridged structures. Stronger quenching is even observed in a 95:5 Tb/Eu Tb(NO_3_)_3_ + Eu(NO_3_)_3_ solution complexed with 6 equiv HDEHP shown in Figure 9b.

The differences in these energy transfer observations suggest that there are two distinct Eu^3+^ species present in the resin samples. Direct Eu^3+^ excitation (λ_ex_ = 394 nm) results in emission that is dominated by the Eu^3+^ species sorbed onto the resin. Indirect Eu^3+^ excitation results in emission from Eu^3+^ incorporated into an [EuTb(DEHP)_6_]_*n*_ species. Strong Tb^3+^ emission suggests that only a small proportion of the Tb^3+^ and Eu^3+^ ions form these bridged species in the presence of the resin. Measurements of the Eu^3+^ lifetime on the resin samples should result in a biexponential decay to confirm the presence of two Eu^3+^ species; however, the emission intensities in this system were too weak to reliably measure accurate lifetimes.

In order for the oligomeric O–P–O-bridged Ln_2_(DEHP)_6_ species to be present on the resin, it is likely that some HDEHP has been released from the polymer support; HDEHP leaching from LN resin is likely as it is not covalently bound to the polymer support. Quantification of the mass of HDEHP leached in each respective fraction was carried out by TRISKEM using inductively coupled plasma mass spectroscopy (see Supporting Information, Table S2); it was calculated that up to 390 μg of HDEHP (0.17% of total mass on resin), leaches from the polymer support over the course of one LN resin column. Leached HDEHP is now available to form the bridged polymeric structure characterized under aqueous conditions, resulting in the two Eu^3+^ species measured with luminescence of the resin. If all available leached HDEHP formed an extended polymeric network, then 4% of the total carriers could be complexed in the proposed Ln_2_(DEHP)_6_ structure. The elution of the 4% of lanthanides on the column could now be altered by the bridging O–P–O moiety and eluting past their expected concentration; hence, only a small amount of Sm^3+^ and Eu^3+^ coelution is observed.

## 4 Conclusions

The speciation of HDEHP with trivalent lanthanides in solution and the solid state has been investigated in order to determine the species responsible for Sm^3+^ and Eu^3+^ leaching into Tb^3+^ fractions in LN resin, which is routinely used to separate these ions from one another in a number of separation applications including quantification, rare earth recycling and recovery, and nuclear medicine. Extensive spectroscopic measurements have shown that the coordination between HDEHP and trivalent lanthanides in aqueous conditions is a pseudo-octahedral arrangement of O atoms around Ln^3+^ centers bridged through the deprotonated O–P–O moiety in an oligomeric species [Ln_2_(DEHP)_6_]_*n*._ The rise time for Eu^3+^ emission when excited via Tb^3+^ excited states matched the decay of Tb^3+^, confirming that lanthanide centers are brought close enough together in the bridged structures for energy transfer to occur, which is rate-limiting on the Tb^3+^ excited state manifold decay. The O–P–O bridging of different lanthanide centers was shown to affect the concentration at which DEHP^–^ was protonated and the lanthanide released from the complex. Luminescence measurements of lanthanide soaked LN resin showed energy transfer from Tb^3+^ to Eu^3+^, confirming that bridged species are present in a small concentration along with Ln^3+^ sorbed directly onto the resin caused by bleeding of HDEHP from the polymeric resin support. The presence of these bridged complexes when the separations are performed is likely to result in the coelution of Eu^3+^ and Sm^3+^ caused by the presence of Y^3+^ and Tb^3+^. Future investigations will aim to disrupt the formation of these structures on the resin and study how this affects separation behavior. These findings indicate that the stability of LN resin and all other resin types where the extractant is not chemically bound to the polymeric support should be assessed for leaching of the extractant, since it could significantly alter the separation performance achieved by such resins.

## Supporting Information Available

Infrared spectroscopic data of all compounds, CHN data for all compounds, MALDI mass spectrometric data for all compounds, emission spectra and lifetime traces for HDEHP lanthanide titrations and isolated compounds, luminescence spectra as a function of nitric acid concentration, and HDEHP concentrations as a function of nitric acid resin leaching experimental data (PDF)
