# Supplementary material for: Oligomer Formation Effects on the Separation of Trivalent Lanthanide Fission Products
Source: Inorg Chem. 2024 Jul 10;63(29):13380–91. doi: 10.1021/acs.inorgchem.4c01272 (PMC11270979; doi:10.1021/acs.inorgchem.4c01272)
Supplement: Supplementary file 1 — ic4c01272_si_001.pdf [file ic4c01272_si_001.pdf]

Supplementary Material

Oligomer Formation Effects on the Separation of Trivalent Lanthanide Fission Products

Lauren E. Walker [a], Scott L. Heath [b], Jun Jiang [c], Louise S. Natrajan [a,*], and Francis R. Livens [a,*]

[a] *Department of Chemistry, Faculty of Science and Engineering, The University of Manchester, Manchester M13 9PL, UK*
[b] *Department of Earth and Environmental Sciences, The University of Manchester, Manchester M13 9PL, UK*
[c] *AWE, Aldermaston RG7 4PR, UK*
** Corresponding authors. Email addresses:* louise.natrajan@manchester.ac.uk (L. S. Natrajan), francis.livens@manchester.ac.uk (F. R. Livens).

Table of Contents

1. Infrared data **2**

Figure S1 2,3

2. CHN data **3**

Table S1 3

3. Mass spectrometry analysis **4**

Figure S2 4

Figure S3 5,6

Figure S4 6,7

Figure S5 8

Figure S6 8

4. Luminescence HDEHP titrations **9**

Figure S7 9

Figure S8 10

5. Lifetime traces **11**

Figure S9 11

Figure S10 11

Figure S11 11

Figure S12 12

Figure S13 12

Figure S14 12

Figure S15 13

6. Luminescence nitric acid titrations **14**

Figure S16 14

7. HDEHP leaching from LN resin **15**

Table S2 15

1. Infrared data

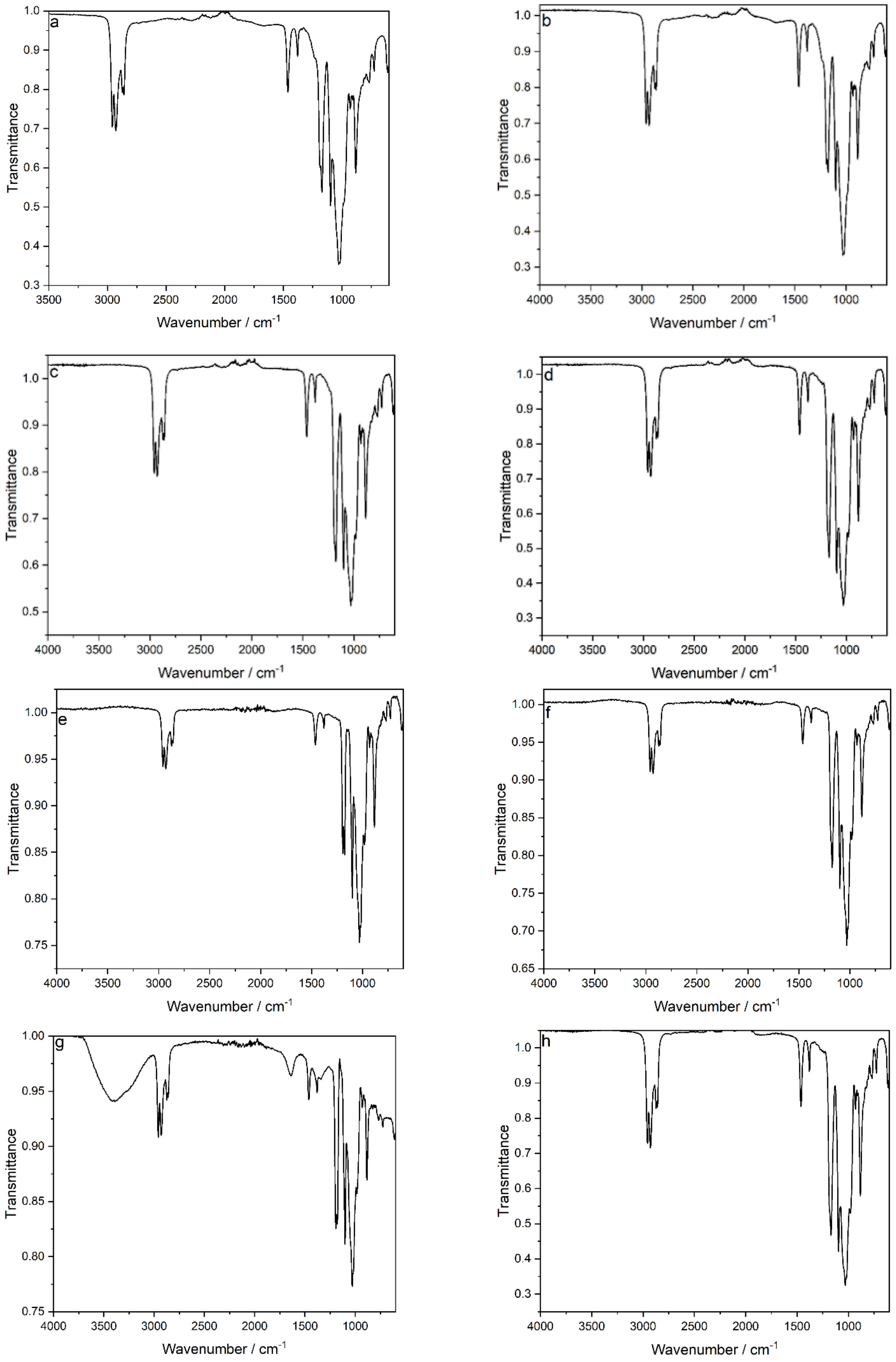

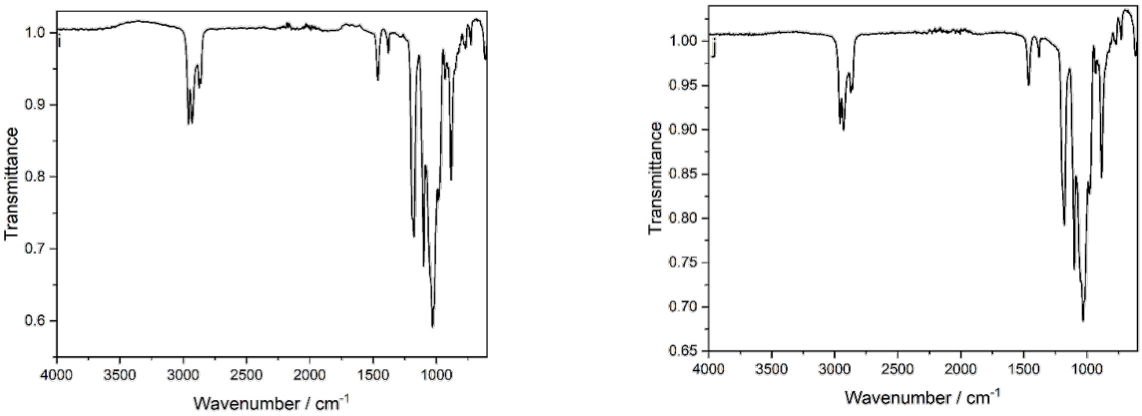

Figure S1. Infrared spectrum recorded of solids collected from the extraction of Ln^{3+} by HDEHP at an aqueous organic interface. **a)** Ln = Eu, **b)** Ln = Eu + Tb, **c)** Ln = Eu + Y, **d)** Ln = Eu + Sm, **e)** Ln = Tb, **f)** Ln = Tb + Sm, **g)** Ln = Tb + Y, **h)** Ln = Sm, **i)** Ln = Sm + Y, **j)** Ln = Y.

2. CHN data

Table S1. CHN analysis of Ln(HDEHP) solids, with expected values for an $Ln(HDEHP)_3$ species. Ln^{3+} content was determined with ICP-AES (inductively coupled plasma atomic emission spectroscopy) analysis.

Ln^{3+}	C %	C% expected	H %	H% expected	Ln %		Ln % predicted (for 1:1 complexes)	
Eu	51.51	51.65	9.36	9.21	Eu: 13.54	-	Eu: 13.61	-
Tb	51.27	51.33	9.47	9.15	Tb: 13.92	-	Tb: 14.15	-
Sm	51.73	51.72	9.17	9.22	Sm: 13.12	-	Sm: 13.49	-
Y	54.88	54.74	9.93	9.76	Y: 8.45	-	Y: 8.44	-
Eu, Y	53.64	53.15	9.63	9.48	Eu: 4.84	Y: 5.23	Eu: 7.00	Y: 4.10
Tb, Y	53.17	52.98	9.70	9.45	Tb: 6.64	Y: 4.45	Tb: 7.30	Y: 4.09
Sm, Y	53.28	53.19	9.43	9.49	Sm: 4.15	Y: 5.72	Sm: 6.94	Y: 4.10
Eu, Tb	51.32	51.49	9.20	9.18	Eu: 5.17	Tb: 8.29	Eu: 6.79	Tb: 7.10
Eu, Sm	51.57	51.69	9.30	9.22	Eu: 7.48	Sm: 5.75	Eu: 6.81	Sm: 6.74
Tb, Sm	51.59	51.53	9.44	9.19	Tb: 9.45	Sm: 4.41	Tb: 7.10	Sm: 6.72

3. Mass spectrometry analysis

Predicted m/z plots for each complex were created using the mMass software. The predicted spectra were further manipulated using Origin Pro in order to be scaled according to the relative concentration of aggregates with different lanthanide ratios. This scaling was carried out using an iterative process where the best fit was achieved through visual inspection and residual fitting.

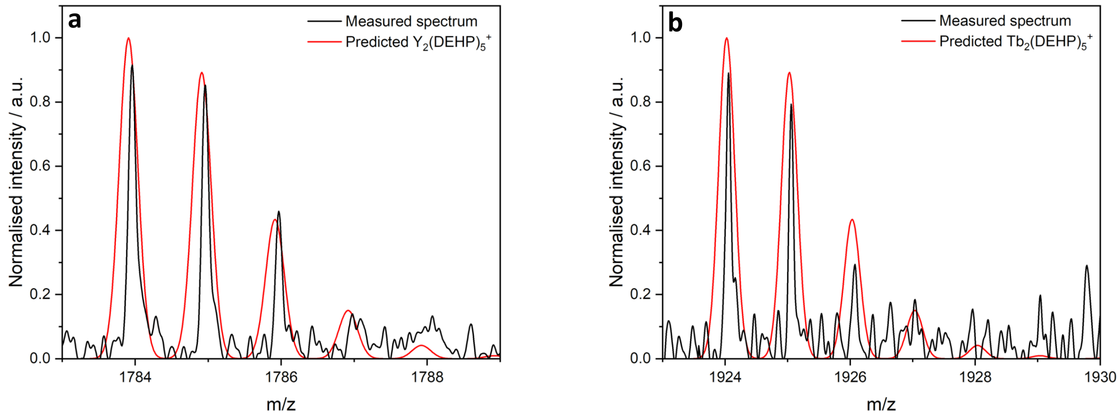

Figure S2. MALDI spectrum showing molecular ion peak of $Ln_2(DEHP)_5^+$ for solids collected from extraction of Ln^{3+} by HDEHP at an aqueous organic interface. **a)** Ln = Y **b)** Ln = Tb, all predicted spectra in red.

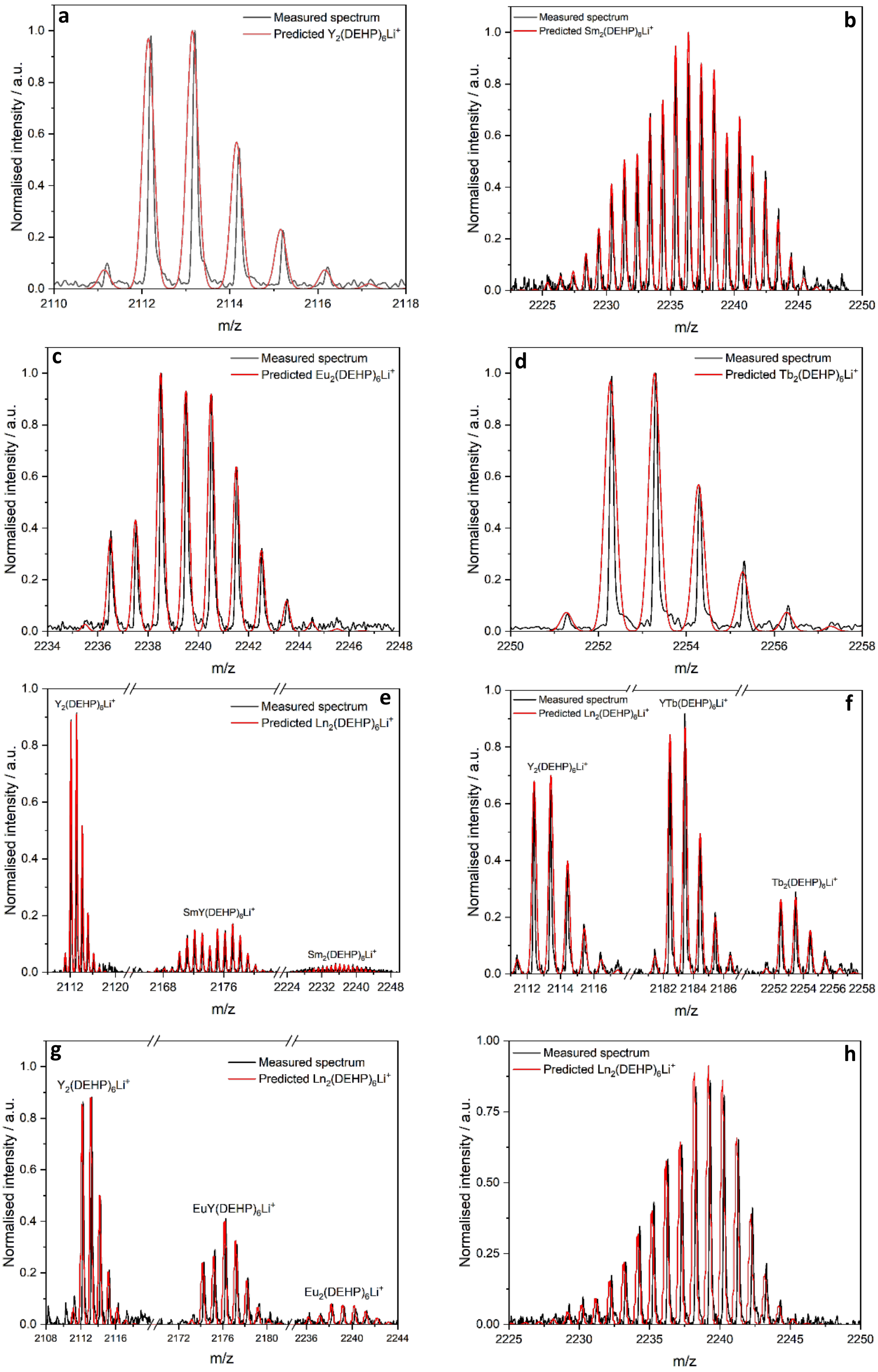
a
Measured spectrum
Predicted Y2(DEHP)6Li+
Normalised intensity / a.u.
m/z
b
Measured spectrum
Predicted Sm2(DEHP)6Li+
c
Predicted Eu2(DEHP)6Li+
d
Predicted Tb2(DEHP)6Li+
e
Predicted Ln2(DEHP)6Li+
Y2(DEHP)6Li+
SmY(DEHP)6Li+
Sm2(DEHP)6Li+
f
YTb(DEHP)6Li+
Tb2(DEHP)6Li+
g
EuY(DEHP)6Li+
Eu2(DEHP)6Li+
h

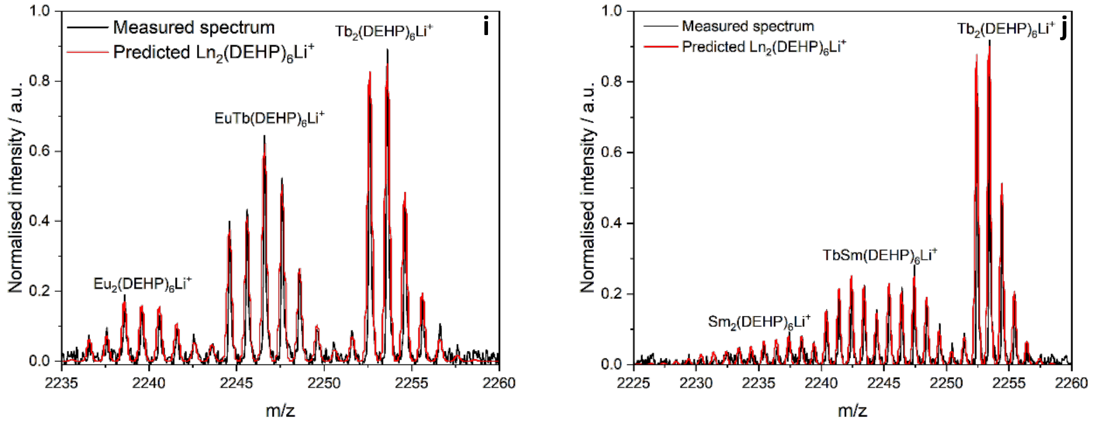

Figure S3. MALDI spectrum showing molecular ion peak of $Ln_2(DEHP)_6Li^+$ for solids collected from extraction of Ln^{3+} by HDEHP at an aqueous organic interface. **a)** Ln = Y **b)** Ln = Sm **c)** Ln = Eu **d)** Ln = Tb **e)** Ln = Sm + Y **f)** Ln = Tb + Y **g)** Ln = Eu + Y **h)** Ln = Sm + Eu **i)** Ln = Eu + Tb **j)** Ln = Tb + Sm, predicted spectra for all in red.

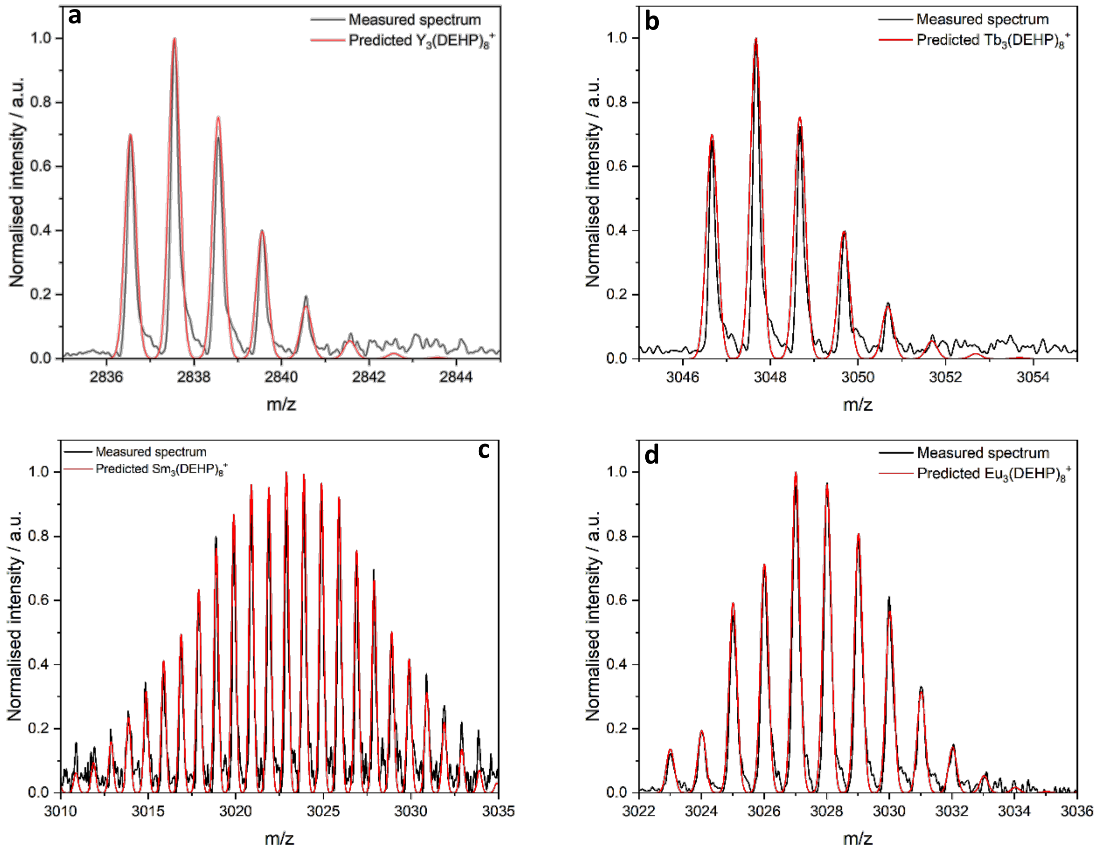

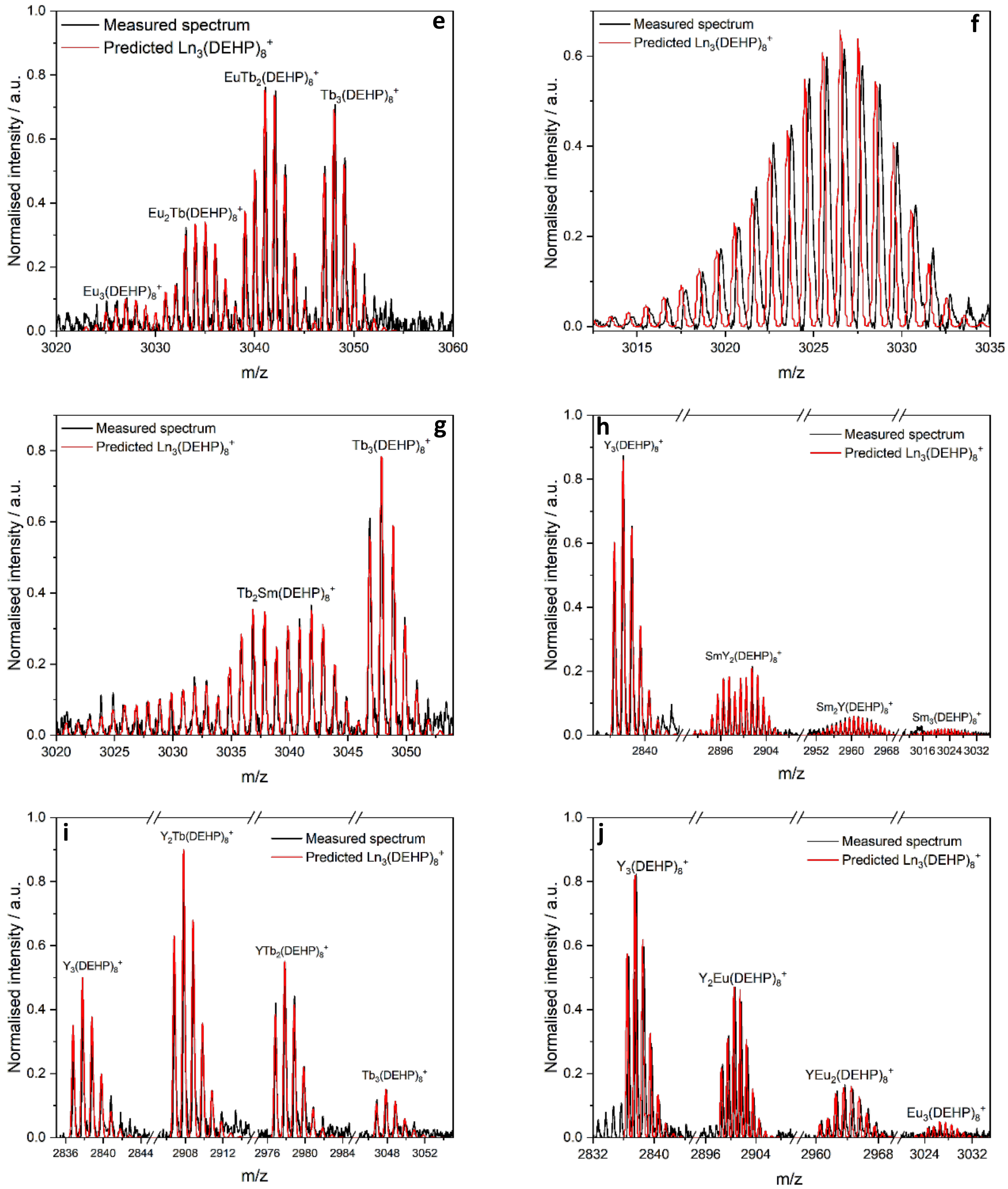

Figure S4. MALDI spectrum showing molecular ion peak of $Ln_3(DEHP)_8^+$ for solids collected from extraction of Ln^{3+} by HDEHP at an aqueous organic interface. **a)** Ln = Y **b)** Ln = Tb **c)** Ln = Sm **d)** Ln = Eu **e)** Ln = Eu + Tb **f)** Ln = Sm + Eu **g)** Ln = Tb + Sm **h)** Ln = Sm + Y **i)** Ln = Tb + Y **j)** Ln = Eu + Y, all predicted spectra in red.

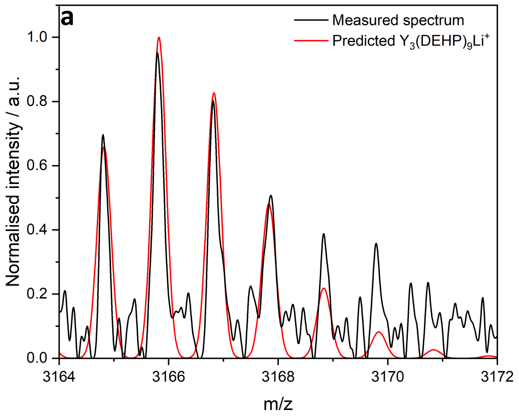

Figure S5. MALDI spectrum showing molecular ion peak of $Ln_3(DEHP)_9Li^+$ for solids collected from extraction of Ln^{3+} by HDEHP at an aqueous organic interface. **a)** Ln = Y **b)** Ln = Tb, all predicted spectra in red.

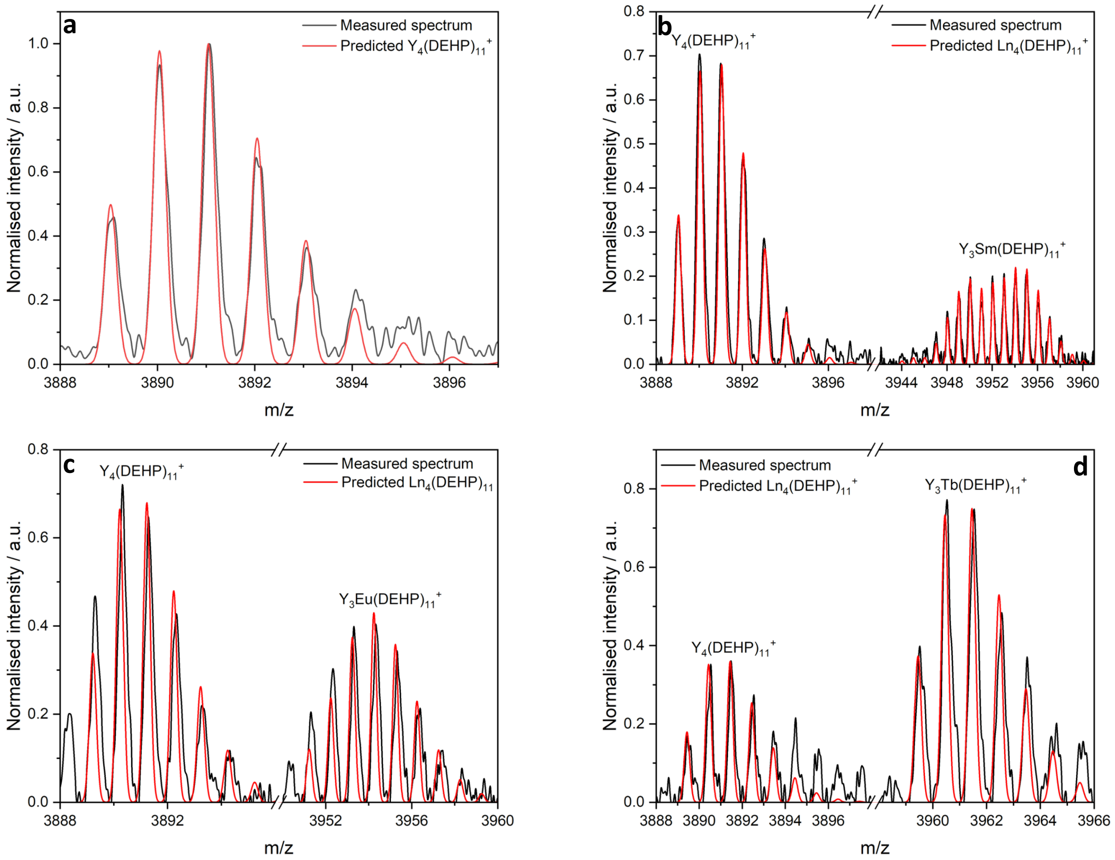

Figure S6. MALDI spectrum showing molecular ion peak of $Ln_4(DEHP)_{11}^+$ for solids collected from extraction of Ln^{3+} by HDEHP at an aqueous organic interface. **a)** Ln = Y**b)** Ln = Eu + Y **c)** Ln = Tb + Y **d)** Ln = Sm + Y, predicted spectra for all in red.

4. Luminescence HDEHP titrations

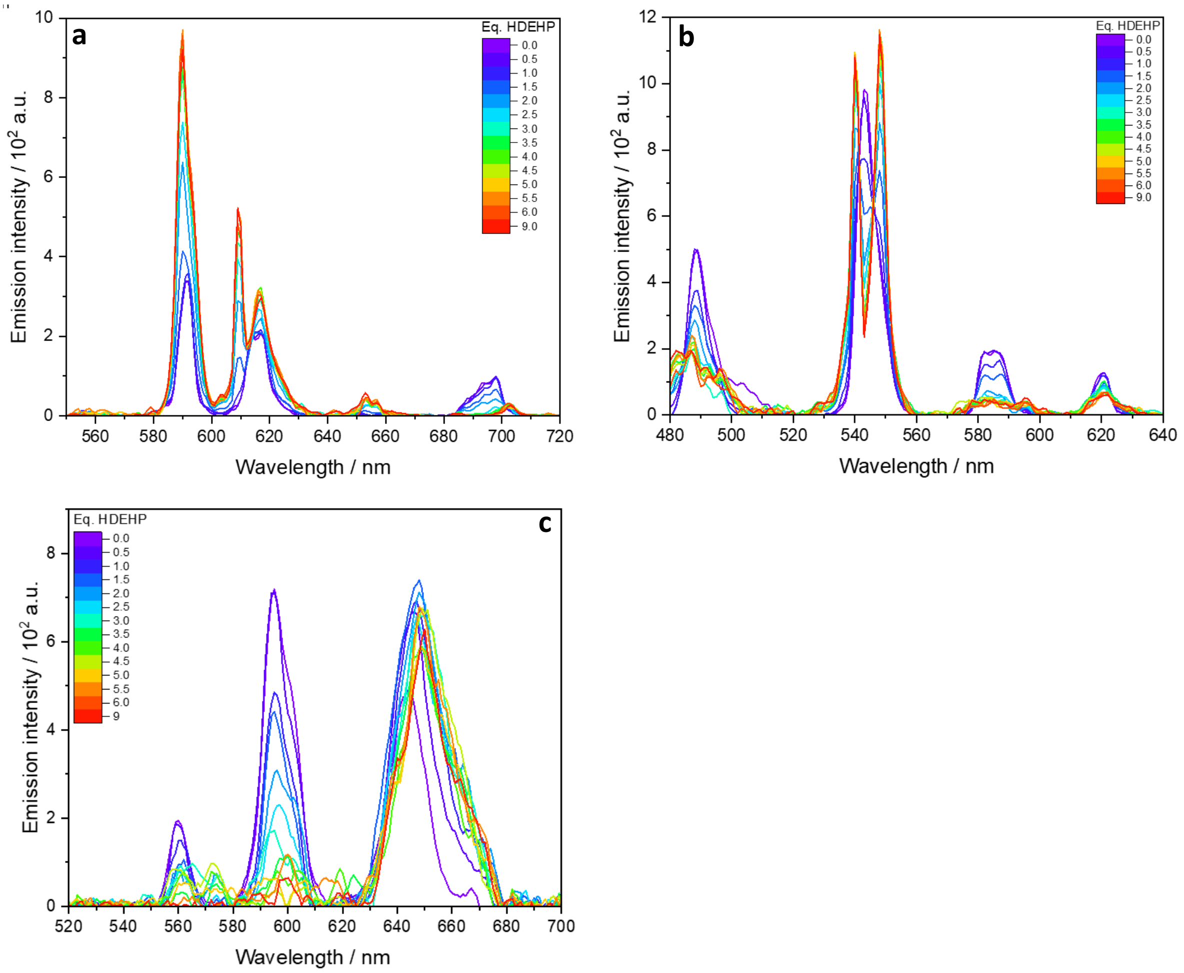

Figure S7. Luminescence emission spectrum of 5 mM **a)** $Eu(NO_3)_3$, λ_{ex} = 394 nm **b)** $Tb(NO_3)_3$, λ_{ex} = 369 nm **c)** $Sm(NO_3)_3$, λ_{ex} = 402 nm titrated with HDEHP up to 6 molar equivalents in 0.5 molar equivalent intervals, final spectra measured with 9 molar equivalents HDEHP. All measured in a 1:1 v:v H_2O:IPA solvent mixture.

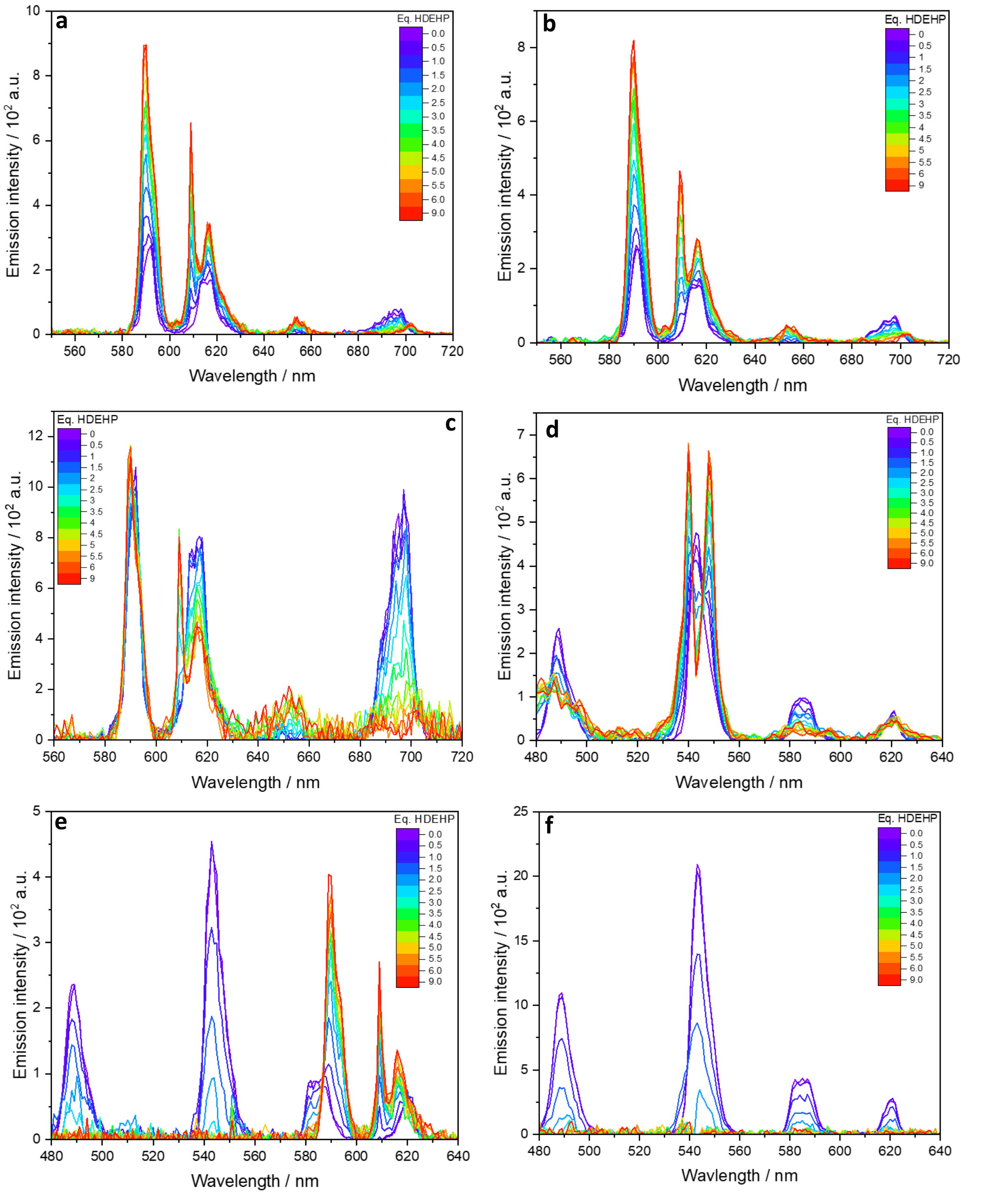

Figure S8. Luminescence emission spectrum of 5 mM **a)** 1:1 $Eu(NO_3)_3$ + $Y(NO_3)_3$, λ_{ex} = 394 nm **b)** 1:1 $Eu(NO_3)_3$ + $Tb(NO_3)_3$, λ_{ex} = 394 nm **c)** 1:1 $Eu(NO_3)_3$ + $Sm(NO_3)_3$, λ_{ex} = 394 nm, **d)** 1:1 $Tb(NO_3)_3$ + $Y(NO_3)_3$, λ_{ex} = 369 nm **e)** 1:1 $Tb(NO_3)_3$ + $Eu(NO_3)_3$, λ_{ex} = 369 nm **f)** 1:1 $Tb(NO_3)_3$ $Sm(NO_3)_3$, λ_{ex} = 394 nm titrated with HDEHP up to 6 molar equivalents in 0.5 molar equivalent intervals, final spectra measured with 9 molar equivalents HDEHP. All measured in a 1:1 v:v H_2O:IPA solvent mixture.

5. Lifetime traces

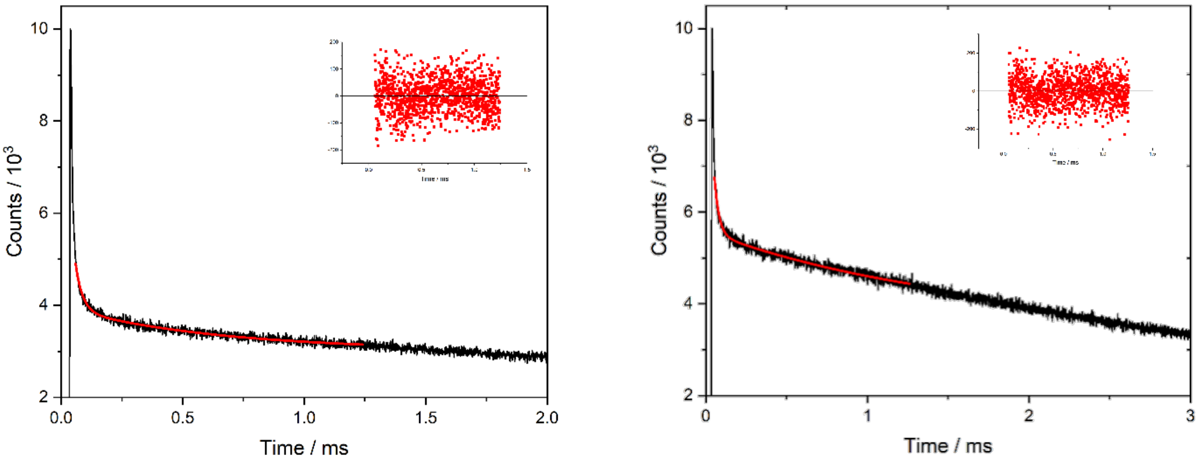

Figure S9. Luminescence decay profile of the Eu^{3+} emission of $[Eu_2(DEHP)_6]_n$ (black) with corresponding mono-exponential fit (red) (λ_{ex} = 394 nm, λ_{em} = 610 nm) in (left) H_2O + IPA, (right) D_2O + d-IPA. Insets show residual of expected error variance for the exponential fit.

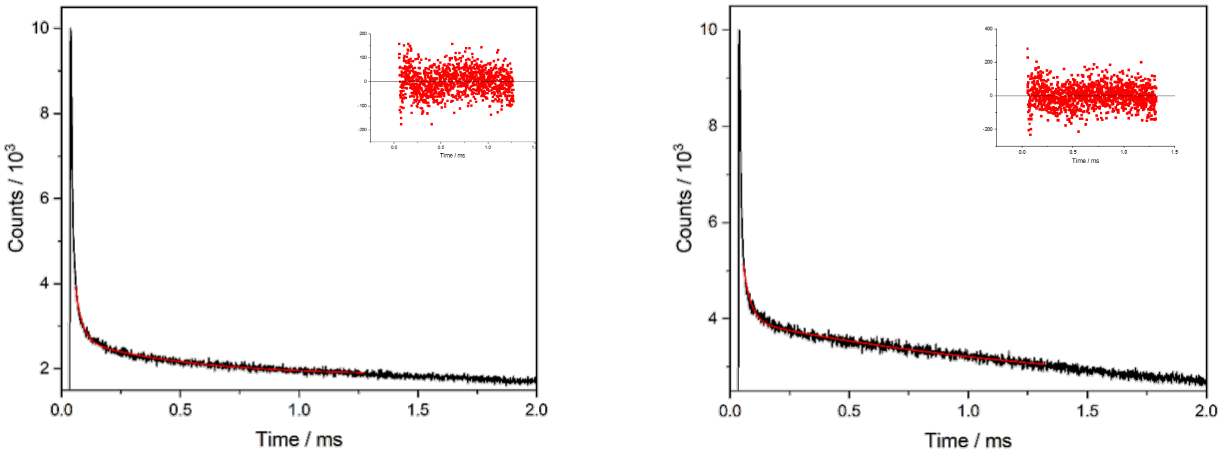

Figure S10. Luminescence decay profile of the Eu^{3+} emission of $[EuTb(DEHP)_6]_n$ (black) with corresponding mono-exponential fit (red) (λ_{ex} = 394 nm, λ_{em} = 610 nm) in (left) H_2O + IPA, (right) D_2O + d-IPA. Insets show residual of expected error variance for the exponential fit.

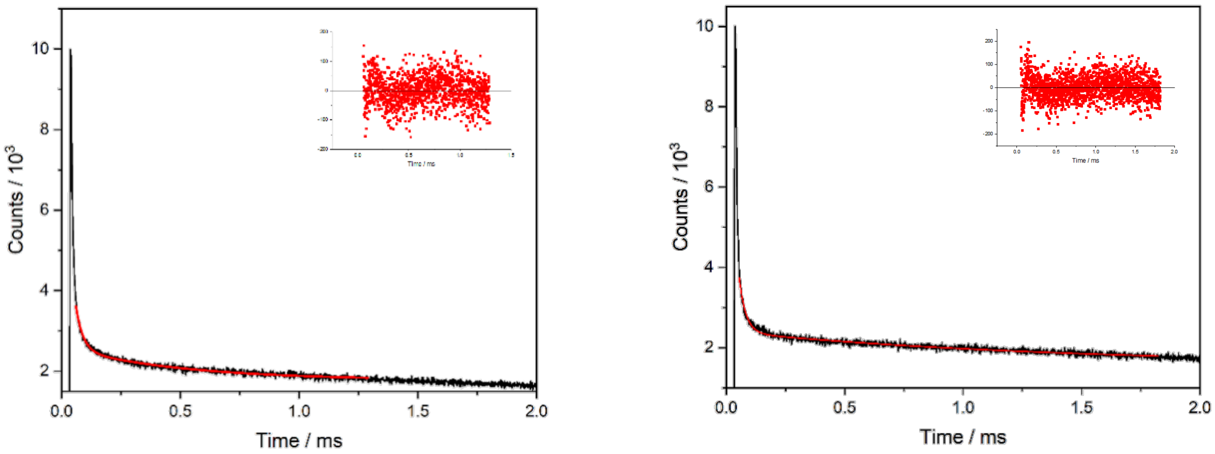

Figure S11. Luminescence decay profile of the Eu^{3+} emission of $[EuSm(DEHP)_6]_n$ (black) with corresponding mono-exponential fit (red) (λ_{ex} = 394 nm, λ_{em} = 610 nm) in (left) H_2O + IPA, (right) D_2O + d-IPA. Insets show residual of expected error variance for the exponential fit.

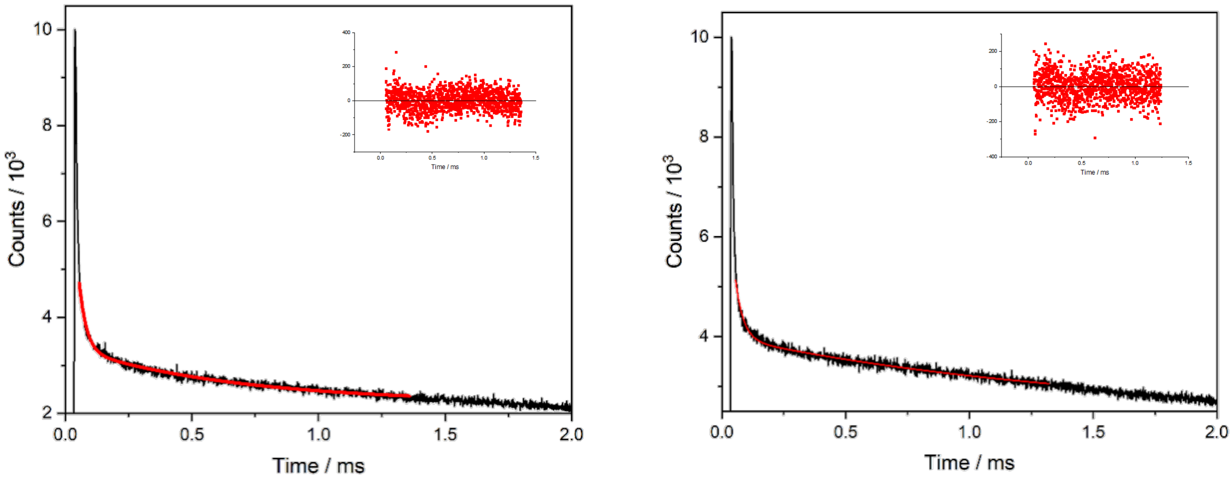

Figure S12. Luminescence decay profile of the Eu^{3+} emission of $[EuY(DEHP)_6]_n$ (black) with corresponding mono-exponential fit (red) (λ_{ex} = 394 nm, λ_{em} = 610 nm) in (left) H_2O + IPA, (right) D_2O + d-IPA. Insets show residual of expected error variance for the exponential fit.

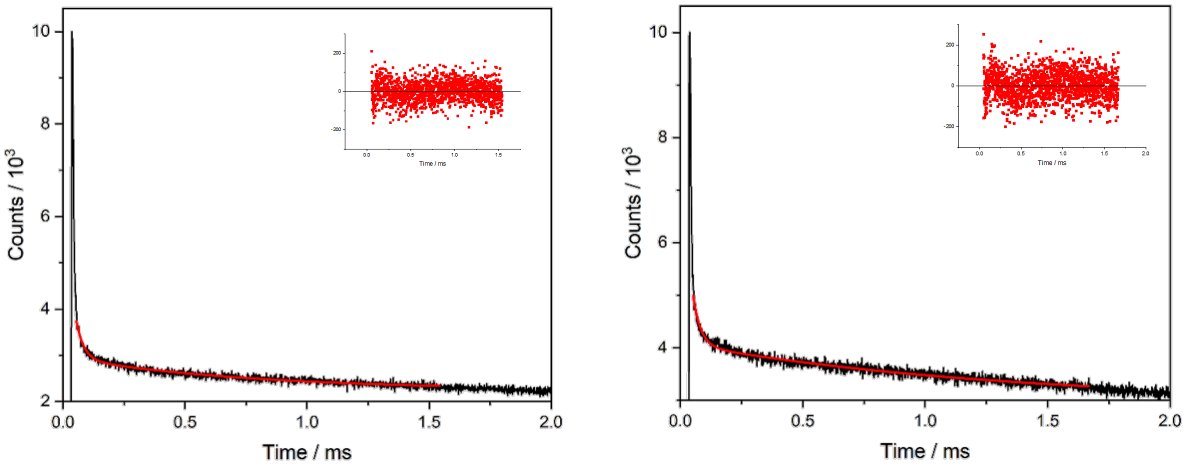

Figure S13. Luminescence decay profile of the Tb^{3+} emission of $[Tb_2(DEHP)_6]_n$ (black) with corresponding mono-exponential fit (red) (λ_{ex} = 369 nm, λ_{em} = 548 nm) in (left) H_2O + IPA, (right) D_2O + d-IPA. Insets show residual of expected error variance for the exponential fit.

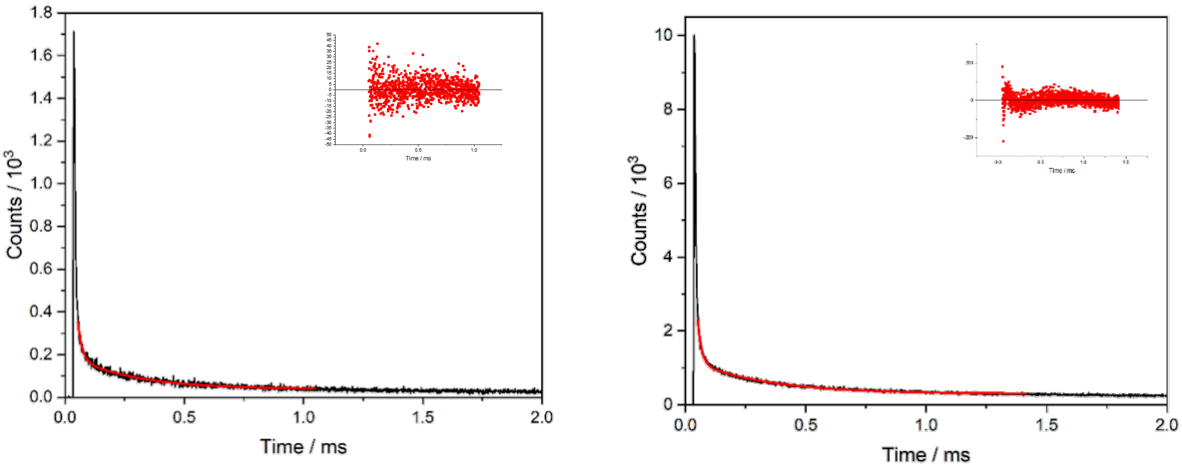

Figure S14. Luminescence decay profile of the Tb^{3+} emission of $[TbEu(DEHP)_6]_n$ (black) with corresponding mono-exponential fit (red) (λ_{ex} = 369 nm, λ_{em} = 548 nm) in (left) H_2O + IPA, (right) D_2O + d-IPA. Insets show residual of expected error variance for the exponential fit.

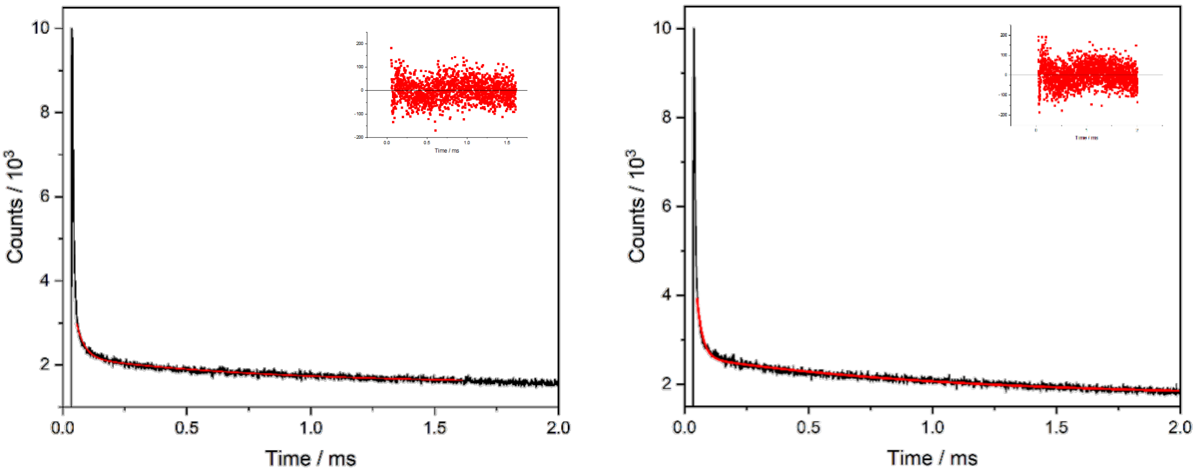

Figure S15. Luminescence decay profile of the Tb^{3+} emission of $[TbY(DEHP)_6]_n$ (black) with corresponding mono-exponential fit (red) (λ_{ex} = 369 nm, λ_{em} = 548 nm) in (left) H_2O + IPA, (right) D_2O + d-IPA. Insets show residual of expected error variance for the exponential fit.

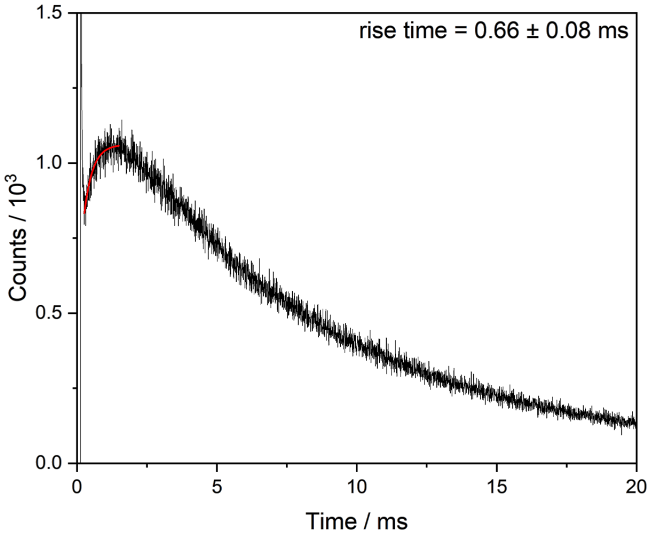

Figure S16. Kinetic trace of $EuTb(HDEHP)_6$ solid Eu^{3+} $^5D_0 \rightarrow ^7F_1$ emission at 590 nm, with Tb^{3+} excitation via the $^7F_6 \rightarrow ^5D_3$ transition at 369 nm, in the solid state showing the slow rise in the emission fitted using an exponential rise function followed by single exponential decay, fitted after subtraction of the instrument response function (IRF)

6. Luminescence nitric acid titrations

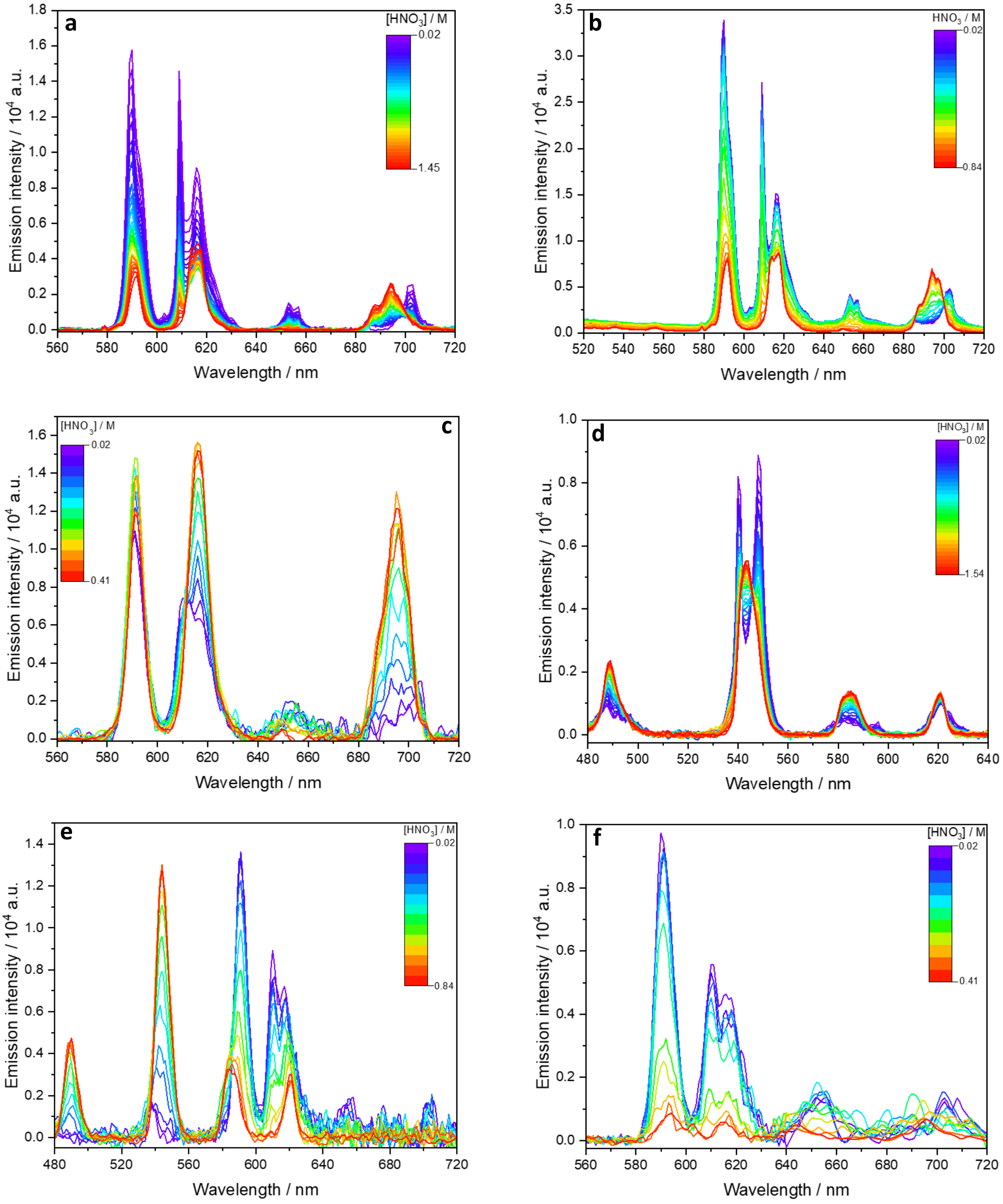

Figure S17. Luminescence emission spectrum of 5 mM **a)** $EuY(DEHP)_6$ λ_{ex} = 394 nm, initial $[HNO_3]$ = 0.02 M, final $[HNO_3]$ = 1.45 M increasing in 0.05 M increments. **b)** $EuTb(DEHP)_6$ λ_{ex} = 394 nm, initial $[HNO_3]$ = 0.02 M, final $[HNO_3]$ = 0.84 M increasing in 0.04 M increments. **c)** $EuSm(DEHP)_6$ λ_{ex} = 394 nm, initial $[HNO_3]$ = 0.02 M, final $[HNO_3]$ = 0.41 M increasing in 0.02 M increments. **d)** $TbY(DEHP)_6$ λ_{ex} = 369 nm, initial $[HNO_3]$ = 0.02 M, final $[HNO_3]$ = 1.54 M increasing in 0.05 M increments. **e)** $TbEu(DEHP)_6$ λ_{ex} = 369 nm, initial $[HNO_3]$ = 0.02 M, final $[HNO_3]$ = 0.84 M increasing in 0.04 M increments. **f)** $SmEu(DEHP)_6$ λ_{ex} = 402 nm, initial $[HNO_3]$ = 0.02 M, final $[HNO_3]$ = 0.41 M increasing in 0.02 M increments. All measured in a 1:1 H_2O:IPA solvent mixture.

7. HDEHP leaching from LN resin

Table S2. Mass of HDEHP contained in eluent from LN resin column, measured after each nitric acid concentration elution using a standard separation scheme.[1] The concentration of HDEHP was determined by TRISKEM through measuring the non-purgeable organic carbon in each nitric acid fraction in the typical scheme used for separations. 8M HNO_3 is close to the limit of suitable conditions for these measurements, resulting in a high uncertainty in the measurements at a high nitric acid concentration.

Fraction	$[HNO_3]$ / M	Mass HDEHP / µg	Error / µg
Sm^{3+}	0.50	41.0	0.5
Eu^{3+}	0.75	34.3	0.2
Tb^{3+}	2.00	44.9	0.2
Tb^{3+}	2.50	59.7	0.6
Y^{3+}	3.00	72.3	1.0
Y^{3+}	8.00	136.1	32.7
Total mass		388.3	

References

1. Jiang, J.; Davies, A.; Arrigo, L.; Friese, J.; Seiner, B. N.; Greenwood, L.; Finch, Z. Analysis of ^{161}Tb by Radiochemical Separation and Liquid Scintillation Counting. *Applied Radiation and Isotopes*, **2021**, 170, 107298. DOI: 10.1016/j.apradiso.2015.12.004.
